# A theoretical and computational study of heteroclinic cycles in Lotka–Volterra systems

**DOI:** 10.1007/s00285-025-02190-4

**Published:** 2025-02-08

**Authors:** M. C. Bortolan, P. Kalita, J. A. Langa, R. O. Moura

**Affiliations:** 1 Departamento de Matemática, Centro de Ciências Físicas e Matemt́icas, Universidade Federal de Santa Catarina, Campus Florianópolis, Florianópolis, SC CEP 88040-090 Brazil; 2 Faculty of Mathematics and Computer Science, Jagiellonian University, ul. Łojasiewicza 6, 30-348 Kraków, Poland; 3 Departamento de Ecuaciones Diferenciales y Análisis Numérico, Universidad de Sevilla, Campus Reina Mercedes, 41012 Sevilla, Spain; 4 Instituto de Ciências Matemáticas e de Computação, Universidade de São Paulo, Campus de São Carlos, São Carlos, SP Caixa Postal 668 Brazil

**Keywords:** Cycle structure, Lotka–Volterra, Volterra–Lyapunov matrix, Alpha-limits, Invasion graph, 37C20, 37M22, 37N25, 34D30

## Abstract

In general, global attractors are composed of isolated invariant sets and the connections between them. This structure can possibly be highly complex, encompassing attraction basins, repeller sets and invariant sets that, collectively, form a dynamical landscape. Lotka–Volterra systems have long been pivotal as preliminary models for dynamics in complex networks exhibiting pairwise interactions. In scenarios involving Volterra–Lyapunov (VL) stable matrices, the dynamics is simplified in such a way that the positive solutions converge to a single, globally asymptotically stable stationary point as time tends to infinity, thereby excluding the existence of periodic solutions. In this work, we conduct a systematic study on the emergence of heteroclinic cycles within Lotka–Volterra systems characterized by Volterra–Lyapunov stable matrices. Although VL stability of the matrix implies that $\omega$-limit sets of solutions are always stationary points, our analysis of $\alpha$-limit sets reveals finite sets of stationary points interconnected by global trajectories, forming structures referred to as *heteroclinic cycles*. Our findings indicate that even within the framework of VL stable matrices, such structures are more prevalent than previously thought in literature, driven by the interplay between the symmetric and antisymmetric components of the model matrix. This understanding also reinforces our comprehension of the classical three-dimensional May–Leonard model, which is known to be the unique case exhibiting heteroclinic cycle within the VL framework in dimension three, while also pointing to a surprising richness in the dynamics of these structures in higher dimensions.

## Introduction

One of the most significant problems in modern dynamical systems theory is to describe in detail the structure of the invariant sets of a system. The largest of the bounded invariant sets for dissipative models is the *global attractor* (Babin and Vishik 1992; Hale 1988; Henry 1981; Ladyzhenskaya 1991; Robinson 2001; Temam 1997), which determines the dynamics of a phenomenon and has the capability to describe the entire behavior, both transient and asymptotic, of the associated solutions. Additionally, when we can characterize the geometric structure of the global attractor in a model governed by ordinary or partial differential equations, we obtain the complete qualitative information of the phenomenon. The study of this structure has received much attention in recent decades (Aragão-Costa et al. 2011; Babin and Vishik 1992; Hale et al. 2002; Carvalho et al. 2013; Joly and Raugel 2010), despite the difficulties in obtaining a full description of these sets. A global attractor is generically composed of isolated invariant sets and connections between them (Carvalho et al. 2013; Robinson 2001; Bortolan et al. 2020), allowing us to divide the states within this set into recurrent and gradient points (Conley 1978; Norton 1995). Recurrent points are those where the dynamics returns repeatedly through finite chains of solutions and are typically related to chaotic dynamics, very sensitive to initial conditions; on the other hand, gradient points are those through which solutions connect the different invariant sets present in the attractor. These invariant sets are generally composed precisely of recurrent states. As we can observe, the structure of the attractor may possess high complexity, with diverse zones, attraction basins, repeller sets, and invariant sets that create a true landscape of dynamic information (Carvalho et al. 2025).

On the other hand, for many decades, Lotka–Volterra systems have been studied as a first approximation model for dynamics in a complex network of pairwise node interactions. The model reads as:

$$\begin{aligned} \dot{u}_i = u_i\left( b_i + \sum _{j=1}^N a_{ij}u_j\right) \quad \hbox { for } i=1,\ldots ,N, \end{aligned}$$

with $\textbf{b} = (b_1,\ldots ,b_N)\in \mathbb {R}^N$ representing the *intrinsic growth rates* of *N* species and $A = (a_{ij})_{i,j=1}^N \in \mathbb {R}^{N \times N}$ is the associated *interaction matrix*.

One of the cases in which the dynamics appears to be simpler is the case of Volterra–Lyapunov (VL) stable matrices *A* (see Definition 3.1), in which the asymptotic behavior of positive solutions ends in a single stationary point, which is globally asymptotically stable (at least) within the set of strictly positive solutions (Takeuchi 1996). This fact prevents, for example, the existence of periodic solutions for the model. However, as we described above, the global attractor is not primarily composed of attracting invariant sets (such as a globally asymptotically stable stationary points) but, for instance, in the case of gradient systems (Hale 1988; Robinson 2001; Carvalho et al. 2013; Bortolan et al. 2022), the attractor is described as the union of the unstable manifolds associated with the invariant sets, which means that the attractor may possess significant repulsion basins within it. In this work it is precisely this fact of considering the dynamics within the global attractor as time progresses towards minus infinity that makes it possible to detect new invariant sets of recurrent points beyond the stationary points of the system. In this way, we describe the appearance of heteroclinic cycles (Afraimovich et al. 2008; Hofbauer 1994; Guckenheimer and Holmes 1988; Krupa 1997) (also called homoclinic structures Bortolan et al. (2020), Definition 3.16) for Lotka–Volterra systems with VL stable matrices. As we have indicated, this latter hypothesis implies that the $\omega$-limit sets of particular solutions are always stationary points. However, as we detail in this work, the analysis of the $\alpha$-limit sets allows us to observe the emergence of finite sets of stationary points connected by global trajectories forming a cyclic structure. These *recurrent heteroclinic structures*, even in the case of VL stable matrices, are much more common than previously thought in the literature, and are related to, as we demonstrate, a balance between the symmetric and antisymmetric parts of the model matrix (see Sect. 5). Note that the presence of a heteroclinic cycle produces dynamics significantly more complex than in the case of the equilibria connected by heteroclinic solutions in a directed acyclic graph which could lead to a better understanding of real complex phenomena in nature (see May and Leonard (1975)). This is why it is so important to describe in detail the geometrical properties of a global attractor. ln general, due to the presence of invariant subspaces in which the solutions that constitute the cycle lie, these complicated structures may be structurally stable, i.e., they are robust under perturbation (Guckenheimer and Holmes 1988; Lohse 2023; Krupa 1997; Bortolan et al. 2020, 2022). Our observations lead us to revisit a classic May–Leonard model (May and Leonard 1975; Chi et al. 1998) in three dimensions, in which a heteroclinic cycle of the three unique stationary points with one non-zero component appears. While our analysis demonstrates that the May–Leonard model is the only case in three dimensions where we can find such a cycle (Theorem 4.2), for higher dimensions, the dynamical richness of these models will appear surprising, allowing the presence of heteroclinic cycles of arbitrary sizes, between equilibria with a high number of present species, and possibly containing connections between equilibria with different numbers of species.

As we previously mentioned, Volterra–Lyapunov stable matrices are very important when working with Lotka–Volterra models, because of the simpler dynamics in the global attractor. These matrices include diagonally dominant matrices, which in general are expected in ecological systems, where a species has a stronger interaction with itself than with other species. Several results exist to help finding out if a given matrix is Volterra–Lyapunov stable (Kraaijevanger 1991; Redheffer 1985; Cross 1978), and even models not related to ecology can be studied using Volterra–Lyapunov stability (Mayengo 2023; Chien and Shateyi 2021). In this work, we also give a constructive characterization of VL stable matrices, that helps with the task of generating them, and study the associated LV system. In Sect. 5.1, we provide a numerical approach to discover if a given matrix is VL stable or not.

## $\alpha$-limits of semigroups

In this section we discuss important results regarding the behavior and structure of $\alpha$-limits of global solutions for abstract semigroups in metric spaces. To that end, we consider $\mathcal {T}=\{T(t):t\geqslant 0\}$ a **semigroup** in a metric space (*X*, *d*), that is, a family of continuous maps $T(t):X\rightarrow X$ indexed in $t\geqslant 0$, satisfying:

$$\circ $$
$$T(0)x=x$$ for all $$x\in X$$;
$$\circ $$
$$T(t+s)=T(t)T(s)$$ for all $$t,s\geqslant 0$$;
$$\circ $$
the map $[0,\infty )\times X\ni (t,x)\mapsto T(t)x\in X$ is continuous.

When $\mathcal {T}=\{T(t):t\in \mathbb {R}\}$, $T(0)x=x$ for all $x\in X$, $T(t+s)=T(t)T(s)$ for all $t,s\in \mathbb {R}$ and the map $\mathbb {R}\times X\ni (t,x)\mapsto T(t)x\in X$ is continuous, we say that $\mathcal {T}$ is a **group**. In this case, note that *T*(*t*) is invertible and $T(t)^{-1}=T(-t)$ for all $t \in \mathbb {R}$.

For a semigroup $\mathcal {T}$ in *X*, the $\omega$**-limit** of a point $x\in X$ is the set defined by

$$\begin{aligned} \omega (x) = \{y\in X:\hbox { there exists } t_n\rightarrow \infty \hbox { such that } T(t_n)x\rightarrow y\}. \end{aligned}$$

A **global solution** of the semigroup $\mathcal {T}$ is a continuous function $\xi :\mathbb {R}\rightarrow X$ such that $T(t)\xi (s)=\xi (t+s)$ for all $t\geqslant 0$ and $s\in \mathbb {R}$. We say that $\xi$ is **bounded** if the set $\xi (\mathbb {R})$ is bounded in *X*. For a given global solution $\xi$ of $\mathcal {T}$ in *X*, the $\alpha$**-limit of** $\xi$ is the set defined by

$$\begin{aligned} \alpha (\xi ) = \big \{y\in X:\hbox { there exists } s_n\rightarrow -\infty \hbox { such that } \xi (s_n)\rightarrow y\big \}. \end{aligned}$$

To see different, but equivalent, definitions of the $\omega$ and $\alpha$-limits, see Bortolan et al. (2020, Proposition 1.13).

We recall that a subset $\mathcal {A}$ of *X* is called a **global attractor** for the semigroup $\mathcal {T}$ if:

$$\circ $$
$$\mathcal {A}$$ is compact;
$$\circ $$
$\mathcal {A}$ is **invariant**, that is, $T(t)\mathcal {A}=\mathcal {A}$ for all $t\geqslant 0$;
$$\circ $$
$\mathcal {A}$ **attracts bounded subsets of** *X* , that is, given $B\subset X$, a bounded set, we have
  $$\begin{aligned} d_H(T(t)B,\mathcal {A})\rightarrow 0 \quad \hbox { as } t\rightarrow \infty , \end{aligned}$$
  where
  $$\begin{aligned} d_H(U,V)=\sup \limits _{u\in U} \inf \limits _{v\in V}d(u,v) \end{aligned}$$
  denotes the *Hausdorff semidistance* between the nonempty sets $U,V\subset X$.

Clearly, when a semigroup $\mathcal {T}$ has a global attractor $\mathcal {A}$, it is unique. Furthermore, in this case,

$$\begin{aligned} \mathcal {A}=\{\xi (0):\xi \hbox { is a bounded global solution of } \mathcal {T}\}. \end{aligned}$$

We now recall a usual result regarding the properties of both $\alpha$ and $\omega$-limits. For a proof see Bortolan et al. (2020, Lemmas 1.15 and 1.16), for instance.

### Lemma 2.1

If the semigroup $\mathcal {T}$ has a global attractor, then for each bounded global solution $\xi$ of $\mathcal {T}$, its $\alpha$-limit $\alpha (\xi )$ is nonempty, compact, invariant and

$$\begin{aligned} d_H(\xi (s),\alpha (\xi ))\rightarrow 0 \quad \hbox { as } s\rightarrow -\infty . \end{aligned}$$

Also, for each $x\in X$ its $\omega$-limit $\omega (x)$ is nonempty, compact, invariant and

$$\begin{aligned} d_H(T(t)x,\omega (x))\rightarrow 0 \quad \hbox { as } t\rightarrow \infty . \end{aligned}$$

A point $e^*$ is called an **equilibrium** for $\mathcal {T}$ if $T(t)e^*=e^*$ for all $t\geqslant 0$. In the next result we prove that if every $\omega$-limit contains an equilibrium, then so does every $\alpha$-limit.

### Lemma 2.2

Assume that $\mathcal {T}$ is a semigroup with a global attractor $\mathcal {A}$ and a nonempty set of equilibria $\mathcal {E}$. If for each $x\in X$ we have $\omega (x)\cap \mathcal {E}\ne \varnothing$, then for any bounded global solution $\xi$ of $\mathcal {T}$ we have $\alpha (\xi )\cap \mathcal {E}\ne \varnothing$.

### Proof

By Lemma 2.1, $\alpha (\xi )$ is nonempty and we choose any $x\in \alpha (\xi )$. If $x\in \mathcal {E}$ the proof is over. Otherwise, again by Lemma 2.1, since $\alpha (\xi )$ is compact and invariant it must contain $\omega (x)$. Since $\omega (x)\cap \mathcal {E}\ne \varnothing$, the proof is complete. $\square$

A global solution $\xi :\mathbb {R}\rightarrow X$ is called **stationary** if $\xi (t)=e^*$ for all $t\in \mathbb {R}$, where $e^*$ is an equilibrium of $\mathcal {T}$. We say that $\xi$ is **nontrivial** if it is not a stationary global solution.

For $\delta >0$ and $x\in X$, $\overline{B}_\delta (x)$ ($B_\delta (x)$) denotes the closed (open) ball in *X* with radius $\delta$ centered at *x*. We say that an equilibrium $e^*$ of $\mathcal {T}$ is an **isolated equilibrium** if there exists $\delta >0$ such that if $e^*\subset E \subset \overline{B}_\delta (e^*)$ and *E* is an invariant set, then $E=\{e^*\}$. We also say that $e^*$ is the **maximal invariant set in** $\overline{B}_\delta (e^*)$.

### Lemma 2.3

Let $e^*$ be an equilibrium of $\mathcal {T}$, and assume that $e^*$ is the maximal invariant set in $\overline{B}_\delta (e^*)$. If $\xi$ is a global solution of $\mathcal {T}$ in *X* with $d(\xi (t),e^*)\leqslant \delta$ for all $t\leqslant 0$ $(t\geqslant 0)$, then

$$\begin{aligned} \xi (t)\rightarrow e^*\quad \hbox { as } t\rightarrow -\infty \ (t\rightarrow \infty ). \end{aligned}$$

### Proof

We prove only the $t \leqslant 0$ case, and the other follows analogously. Since $\xi (t)\in \overline{B}_\delta (e^*)$ for all $t\geqslant 0$, we have $\alpha (\xi )\subset \overline{B}_\delta (e^*)$. Hence

$$\begin{aligned} e^*\subset \{e^*\}\cup \alpha (\xi ) \subset \overline{B}_\delta (e^*), \end{aligned}$$

and the maximality of $e^*$ shows that $\alpha (\xi )\subset \{e^*\}$. Since, from Lemma 2.1, $\alpha (\xi )\ne \varnothing$, we obtain $\alpha (\xi )=\{e^*\}$. Using again Lemma 2.1, we obtain $\xi (t)\rightarrow e^*$ as $t\rightarrow -\infty$. $\square$

### Lemma 2.4

Let $e^*$ be an equilibrium of $\mathcal {T}$. Given $\ell >0$ and $\varepsilon >0$, there exists $0<\mu <\varepsilon$ such that $T(t)B_\mu (e^*)\subset B_\varepsilon (e^*)$ for all $t\in [0,\ell ]$.

Furthermore, assuming that $\mathcal {T}$ has a global attractor $\mathcal {A}$ and that

2.1 $$\begin{aligned} \hbox { if }x\in \mathcal {A} \hbox { and } T(t)x=e^*\hbox { for some } t>0 \hbox { we have } x=e^*, \end{aligned}$$

then if $d(x_n,e^*)\geqslant \varepsilon$, with $x_n\in \mathcal {A}$ and $\varepsilon >0$, and $t_n\geqslant 0$ is such $T(t_n)x_n\rightarrow e^*$, we have $t_n\rightarrow \infty$.

### Proof

To prove the first assertion, notice, that if that is not the case, there exist $\ell >0$, $\varepsilon >0$, and sequences $x_n\rightarrow e^*$ and $t_n\in [0,\ell ]$ such that $d(T(t_n)x_n,e^*)\geqslant \varepsilon$ for all $n\in \mathbb {N}$. Since $[0,\ell ]$ is compact, up to a subsequence, we can assume that $t_n\rightarrow t_0\in [0,\ell ]$. Hence $T(t_n)x_n\rightarrow T(t_0)e^*= e^*$, which gives us a contradiction.

For the second claim, if some subsequence of $\{t_n\}_{n\in \mathbb {N}}$ is bounded, then up to a subsequence of this subsequence (which we name the same) we have $t_n\rightarrow t_0$ for some $t_0\geqslant 0$. Since $\{x_n\}_{n\in \mathbb {N}}\subset \mathcal {A}$, up to a subsequence we have $x_n\rightarrow x\in \mathcal {A}$. Hence $x\ne e^*$, thus $t_0>0$ and $T(t_0)x=e^*$, which contradicts (2.1). $\square$

Before continuing, we present a slightly simplified version of a crucial result from Bortolan et al. (2020).

### Lemma 2.5

(Bortolan et al. 2020, Lemma 2.4) Assume that $\mathcal {T}$ has a global attractor $\mathcal {A}$. Let $a_n<b_n<c_n$ be such that $b_n-a_n\rightarrow \infty$, $c_n-b_n\rightarrow \infty$, and set $\mathbb {J}_n=[a_n,c_n]$. Let $\xi _n:\mathbb {J}_n\rightarrow X$ be a solution of $\mathcal {T}$ and assume that

$$\begin{aligned} \overline{\cup _{n\in \mathbb {N}} \xi _n(\mathbb {J}_n)} \text{ is } \text{ bounded. } \end{aligned}$$

Then there exists a subsequence $\{\xi _{n_k}\}$ of $\{\xi _n\}$ and a bounded global solution $\phi :\mathbb {R}\rightarrow X$ of $\mathcal {T}$ such that $\xi _{n_k}(t+b_{n_k})\rightarrow \phi (t)$, uniformly for *t* in compact subintervals of $\mathbb {R}$.

With these lemmas we can present the main result of this section.

### Theorem 2.6

Assume that $\mathcal {T}$ has a global attractor $\mathcal {A}$ and let $\xi$ be a bounded global solution of $\mathcal {T}$. Assume also that there exists an isolated equilibrium $e^*\in \alpha (\xi )$, satisfying (2.1), and a point $x\in \alpha (\xi )$, with $x\ne e^*$.

Then there exist nontrivial bounded global solutions $\phi$ and $\psi$ of $\mathcal {T}$ with

$$\begin{aligned} \phi (t) \rightarrow e^*\hbox { as } t\rightarrow -\infty \quad \hbox { and } \quad \psi (t) \rightarrow e^*\hbox { as } t\rightarrow \infty . \end{aligned}$$

Furthermore, $\phi (t),\psi (t)\in \alpha (\xi )$ for all $t\in \mathbb {R}$.

### Proof

We fix $\delta >0$ such that

$$\begin{aligned} \overline{B}_{2\delta }(x) \cap \overline{B}_{2\delta }(e^*)=\varnothing . \end{aligned}$$

We can choose $\delta >0$ sufficiently small so that $e^*$ is the maximal invariant set in $\overline{B}_{\delta }(e^*)$. Hence, there exists a strictly decreasing sequence $\{s_n\}_{n\in \mathbb {N}}$, with $s_n\rightarrow -\infty$, such that

$$\begin{aligned} d(\xi (s_n),e^*) < \tfrac{1}{n} \quad \hbox { for all } n\in \mathbb {N}, \end{aligned}$$

and there exists $\ell _n\in (s_{n+1},s_n)$ such that $d(\xi (\ell _n),e^*) > 2\delta$.

*Claim 1*. $$\ell _n-s_{n+1}\rightarrow \infty $$.

If $\{\ell _n-s_{n+1}\}_{n\in \mathbb {N}}$ is bounded then $\ell _n-s_{n+1}\leqslant \ell$ for some $\ell >0$. Thus, from Lemma 2.4, there exists $\mu >0$ such that $T(t)B_\mu (e^*)\subset B_\delta (e^*)$ for all $t\in [0,\ell ]$. For all *n* large, we have $\xi (s_{n+1})\in B_\mu (e^*)$ and thus $\xi (\ell _n)=T(\ell _n-s_{n+1})\xi (s_n)\in B_\delta (e^*)$, which is a contradiction.

*Claim 2*. $$s_n-\ell _n\rightarrow \infty $$.

Since $T(s_n-\ell _n)\xi (\ell _n) = \xi (s_n)\rightarrow e^*$, and $\xi (\ell _n)\in \mathcal {A}$ is such that $d(\xi (\ell _n),e^*)>2\delta$, this follows directly from Lemma 2.4.

Construction of $\phi$. For each $n\in \mathbb {N}$ we can choose $\tau _n\in [s_{n+1},\ell _n)$ such that

$$\begin{aligned} d(\xi (s),e^*) < \delta \quad \hbox { for } s\in [s_{n+1},\tau _n), \end{aligned}$$

and $d(\xi (\tau _n),e^*) = \delta$. Note that $\tau _n\rightarrow -\infty$, and it follows from Lemma 2.4 that $\tau _n-s_{n+1}\rightarrow \infty$. Using Lemma 2.5, with $a_n=s_{n+1}$, $b_n=\tau _n$ and $c_n=s_n$, we obtain a bounded global solution $\phi$ of $\mathcal {T}$ in $\mathcal {A}$ such that, up to a subsequence,

$$\begin{aligned} \xi (t+\tau _n) \rightarrow \phi (t) \quad \hbox { for all } t\in \mathbb {R}. \end{aligned}$$

Clearly, from the definition of $\alpha (\xi )$, we have $\phi (t)\in \alpha (\xi )$ for all $t\in \mathbb {R}$. Note that for $t=0$ we have

$$\begin{aligned} d(\phi (0),e^*) = \lim _{n\rightarrow \infty }d(\xi (\tau _n),e^*) =\delta , \end{aligned}$$

which means that $\phi (0)\ne e^*$. Moreover, given $t\leqslant 0$, for *n* sufficiently large we have $t+\tau _n \in [s_{n+1},\tau _n)$, which implies that

$$\begin{aligned} d(\xi (t+\tau _n),e^*)<\delta \quad \Rightarrow \quad d(\phi (t),e^*)\leqslant \delta . \end{aligned}$$

This shows that $d(\phi (t),e^*)\leqslant \delta$ for all $t\leqslant 0$. From Lemma 2.3 we obtain

$$\begin{aligned} \phi (t) \rightarrow e^*\quad \hbox { as } t\rightarrow -\infty . \end{aligned}$$

Since $\phi (0)\ne e^*$ and $\phi (t)\rightarrow e^*$ as $t\rightarrow -\infty$ we obtain that $\phi$ is a nontrivial global solution of $\mathcal {T}$.

Construction of $\psi$. For each $n\in \mathbb {N}$ we can choose $\gamma _n\in (\ell _n,s_n]$ such that

$$\begin{aligned} d(\xi (s),e^*)<\delta \quad \hbox { for } s\in (\gamma _n,s_n], \end{aligned}$$

and $d(\xi (\gamma _n),e^*)=\delta$. Note that $\gamma _n\rightarrow -\infty$, and it follows from Lemma 2.4, with $x_n=\xi (\gamma _n)$ and $t_n=s_n-\gamma _n$, that $s_n-\gamma _n\rightarrow \infty$. Using Lemma 2.5, with $a_n = s_{n+1}$, $b_n=\gamma _n$ and $c_n = s_n$, we obtain a bounded global solution $\psi$ of $\mathcal {T}$ in $\mathcal {A}$ such that, up to a subsequence,

$$\begin{aligned} \xi (t+\gamma _n)\rightarrow \psi (t) \quad \hbox { for all } t\in \mathbb {R}. \end{aligned}$$

From the definition of $\alpha (\xi )$, we have $\psi (t)\in \alpha (\xi )$ for each $t\in \mathbb {R}$. For $t=0$ we have

$$\begin{aligned} d(\psi (0),e^*)=\lim _{n\rightarrow \infty }d(\xi (\gamma _n),e^*)=\delta . \end{aligned}$$

For $t\geqslant 0$ given, for *n* large we have $t+\gamma _n \in (\gamma _n,s_n]$ and thus

$$\begin{aligned} d(\xi (t+\gamma _n),e^*)<\delta \quad \Rightarrow \quad d(\psi (t),e^*) \leqslant \delta . \end{aligned}$$

This shows that $d(\psi (t),e^*)\leqslant \delta$ for all $t\geqslant 0$ and from Lemma 2.3 we obtain

$$\begin{aligned} \psi (t)\rightarrow e^*\quad \hbox { as } t\rightarrow \infty . \end{aligned}$$

Since $\psi (0)\ne e^*$ and $\psi (t)\rightarrow e^*$ as $t\rightarrow \infty$ it follows that $\psi$ is a nontrivial global solution of $\mathcal {T}$. $\square$

## *N*-dimensional Lotka–Volterra systems

Consider the *N*-dimensional Lotka–Volterra system

LotV $$\begin{aligned} \dot{u}_i = u_i\left( b_i + \sum _{j=1}^N a_{ij}u_j\right) \quad \hbox { for } i=1,\ldots ,N, \end{aligned}$$

with $\textbf{b} = (b_1,\ldots ,b_N)\in \mathbb {R}^N$ representing the intrinsic growth rates of *N* species and $A = (a_{ij})_{i,j=1}^N \in \mathbb {R}^{N \times N}$ representing their crossed interaction rates. We define the phase space:

$$\begin{aligned} \overline{C}_+ = \{\textbf{x}=(x_1,\ldots ,x_N)\in \mathbb {R}^N:x_i \geqslant 0 \hbox { for } i=1,\ldots ,N\}. \end{aligned}$$

### Volterra–Lyapunov stable matrices

We are particularly interested in Lotka–Volterra systems with matrices *A* that are *Volterra–Lyapunov stable*, accordingly to the following definition (for a more detailed study on matrix stability see Logofet (2005)).

#### Definition 3.1

A real matrix $A \in \mathbb {R}^{N \times N}$ is said to be **Volterra–Lyapunov stable** (**VL stable**, for short) if there exists a matrix $H= \text {diag}(h_i)$ with $h_{i} > 0$ such that $H A+A^{T} H$ is *stable*, that is, all of its eigenvalues are negative. The set of VL stable matrices is denoted in the literature by $S_w$.

The Lotka–Volterra system (LotV), when *A* is VL stable, has several important stability properties that will be stated in this section. We start with a new result of characterization of VL stable matrices.

#### Proposition 3.2

The matrix $A\in \mathbb {R}^{N \times N}$ is VL stable if and only if *A* can be written in the form

$$\begin{aligned} A = H(S + J), \end{aligned}$$

where $H = \text {diag}(h_i)$, with $h_i > 0$ for $i=1,\ldots ,N$, *S* is a symmetric stable matrix in $\mathbb {R}^{N\times N}$, and *J* is an antisymmetric matrix in $\mathbb {R}^{N\times N}$.

#### Proof

If *A* is in this form, it is easy to see that $H^{-1}A + A^TH^{-1} = 2\,S$, which is stable. Hence *A* is VL stable. Conversely, if *A* is VL stable, there exists $H = \text {diag}(h_i)$ with $h_i > 0$, such that $S:=(\frac{H^{-1}}{2})A + A^T (\frac{H^{-1}}{2})$ is stable and symmetric. Thus we can write:

$$\begin{aligned} A = H\left( \frac{H^{-1}}{2}A + A^T \frac{H^{-1}}{2} + \frac{H^{-1}}{2}A - A^T\frac{H^{-1}}{2}\right) = H(S+J) \end{aligned}$$

with $J = (\frac{H^{-1}}{2})A - A^T (\frac{H^{-1}}{2})$, an antisymmetric matrix. $\square$

#### Remark 3.3

The importance of this characterization resides in the fact that it is easy to create (or randomly computationally generate) a symmetric stable matrix *S*, a positive diagonal matrix *H* and an antisymmetric matrix *J*, which enables us to computationally study the possible behaviors we might find in the dynamics of (LotV), when *A* is VL stable. This will be further discussed in Sect. 5.2. The task of determining if a given matrix is VL stable will be explored in Sect. 5.1.

From Almaraz et al. (2024, Theorem 8), we have the following:

#### Proposition 3.4

For each $\textbf{u}_0\in \overline{C}_+$, the problem (LotV) with initial data $\textbf{u}_0$ has a unique globally defined solution $u(t,u_0)$ which is a continuous function of time and the initial data. Hence, (LotV) generates a group $\mathcal {T}=\{T(t):t\in \mathbb {R}\}$ in $\overline{C}_+$ defined by $T(t)\textbf{u}_0=u(t,\textbf{u}_0)$ for each $t\in \mathbb {R}$ and $\textbf{u}_0\in \overline{C}_+$. Moreover, if *A* is VL stable then $\mathcal {T}$ has a global attractor $\mathcal {A}$.

We also make the following definitions:

$$\begin{aligned} C_+ = \operatorname {int}\overline{C}_+ = \{\textbf{x}=(x_1,\ldots ,x_N)\in \mathbb {R}^N:x_i > 0 \hbox { for } i=1,\ldots ,N\}. \end{aligned}$$

If $J\subset \{1,\ldots ,N\}$ we define

$$\begin{aligned} C_+^J = \{\textbf{x}\in \overline{C}_+:x_i>0 \hbox { for } i\in J\}. \end{aligned}$$

Given $\textbf{x}\in \overline{C}_+$, we define $J(\textbf{x}) = \{i\in \{1,\ldots ,N\} :x_i>0\}$ and

$$\begin{aligned} C_+^{J(\textbf{x})} = \{\textbf{y}\in \overline{C}_+:y_i>0 \hbox { for } i\in J(\textbf{x})\}. \end{aligned}$$

For the matrix *A*, given $J=\{i_1,\ldots ,i_m\}\subset \{1,\ldots ,N\}$, with $i_1<i_2<\cdots <i_m$, then the **principal submatrix of** *A* **associated with** *J* is the matrix $A(J)=(a_{i_\ell i_p})_{\ell ,p=1}^m$. Also, for a column matrix $\textbf{b}=(b_i)_{i=1}^N$ we define $\textbf{b}(J)=(b_{i_\ell })_{\ell =1}^m$.

#### Definition 3.5

Assume that *A* is VL stable. The set (which we also call **community**) $I\subset \{1,\ldots ,N\}$ is **admissible** if there exists an equilibrium $\textbf{u}^*=(u^*_1,\ldots ,u^*_N)\in \overline{C}_+$ of (LotV) with $u^*_i>0$ if and only if $i\in I$. The family of all admissible communities will be denoted by $\mathscr {C} \subset 2^{\{1,\ldots ,N\}}$.

We denote the unique equilibrium associated with the admissible community *I* by $\textbf{u}^I$, and if $\textbf{u}^*$ is an equilibrium of (LotV), we denote the associated admissible community by $I(\textbf{u}^*)$.

#### Remark

When the matrix *A* is VL stable, it follows from Takeuchi (1996, Lemma 3.2.1 and Lemma 3.2.2) that for each admissible community $I\subset \{1,\ldots ,N\}$ there exists a unique associated equilibrium $\textbf{u}^I$.

Let $I\in \mathscr {C}$ and $\textbf{u}^I=(u^*_1,\ldots ,u^*_n)$ its associated equilibrium. Following Chesson (1994) and Barabás et al. (2018), we define the **invasion rate** of the species $i\in \{1,\ldots ,N\}$ on the community *I* by

$$\begin{aligned} r_i(I) = b_i + \sum _{j\in I}a_{ij}u_j^*. \end{aligned}$$

We use the convention $r_i(\varnothing ) = b_i$ for each $i\in \{1,\ldots ,N\}$. See Almaraz et al. (2024) for a detailed interpretation of the invasion rates.

Following Hofbauer and Schreiber (2022), we present the construction of the **invasion graph (IG)** and its relations to the *heteroclinic connections* of (LotV).

#### Algorithm 3.6

(Invasion graph - IG) The IG is constructed by the following steps:

**(Step 1)** The set of vertices of IG is $\mathscr {C}$, that is, the vertices are the admissible communities.

**(Step 2)** IG contains the edge from *I* to *J* (denoted by $I \rightarrow J$) if and only if $I \ne J$, $r_j(I) > 0$ for every $j \in J {\setminus } I$, and $r_i(J) < 0$ for every $i \in I \setminus J$.

We now present the definition of the **connection graph (CG)**, which provides direct information about the asymptotic dynamics of (LotV):

#### Definition 3.7

(Connection graph - CG) The set of vertices of the CG is $\mathscr {C}$, that is, the admissible communities. The edge $I\rightarrow J$ exists in the CG if and only if $I\ne J$ and there exists a bounded global solution $\xi$ of (LotV) such that

$$\begin{aligned} \textbf{u}^I {\mathop {\longleftarrow }\limits ^{t \rightarrow -\infty }} \xi (t) {\mathop {\longrightarrow }\limits ^{t \rightarrow \infty }} \textbf{u}^J. \end{aligned}$$

The authors in Almaraz et al. (2024) showed the following relation between IG and CG:

#### Proposition 3.8

- If *A* is VL stable, then IG is a subgraph of CG.
- If $r_i(J) \ne 0$ for $i \notin J$ and $J\in \mathscr {C}$ (that is, all equilibria are hyperbolic), then CG is a subset of IG.

#### Remark

The hyperbolicity of the equilibria stated in item (b) of the proposition above follows from the results of Almaraz et al. (2024, Section 3). In summary, for $J\in \mathscr {C}$, the linearization of the Lotka–Volterra system around $\textbf{u}^J$ is of the form

$$\begin{aligned} w' = \begin{pmatrix} B^{11} & B^{12} \\ 0 & B^{22} \end{pmatrix} w, \end{aligned}$$

where the spectrum of $B^{11}$ lies in $\{\lambda \in \mathbb {C}:\operatorname {Re}\lambda <0\}$, and $B^{22}$ is a diagonal matrix with $B^{22}_{ii} = r_i(J)$. Hence, when all $r_i(J)$ are nonzero, the equilibria are hyperbolic.

It is important to mention that CG represents the dynamical information of (LotV) in its phase space, and is contained inside the global attractor $\mathcal {A}$ of (LotV), when it exists. Meanwhile, IG uses only the invasion rates of (LotV), that can be easily calculated, and still it can be equal to the CG, revealing essential information about $\mathcal {A}$.

To begin with the discussion regarding the dynamical properties of (LotV), from Takeuchi (1996, Theorem 3.2.1) we draw the following result:

#### Proposition 3.9

If *A* is VL stable then for any $\textbf{b}\in \mathbb {R}^N$, there exists a unique equilibrium $\textbf{u}^*\in \overline{C}_+$ of (LotV) which is globally asymptotically stable, in the sense that for each $\textbf{u}_0\in C_+^{J(\textbf{u}^*)}$ we have $T(t)\textbf{u}_0\rightarrow \textbf{u}^*$ as $t\rightarrow \infty$. This $\textbf{u}^*$ is called the GASS (the acronym of Globally Asymptotically Stable Solution) of (LotV).

Moreover, for any set $J=\{i_1,\ldots ,i_m\}\subset \{1,\ldots ,N\}$, with $i_1<i_2<\cdots <i_m$, such that $J(\textbf{u}^*)\subset J$, the point $\textbf{y}\in \mathbb {R}^m$ defined by $y_\ell = u^*_\ell$ for $\ell =1,\ldots ,m$ is the GASS of the *m*-dimensional (LotV) with *A*(*J*) and $\textbf{b}(J)$ replacing *A* and $\textbf{b}$, respectively.

From now on, unless clearly stated otherwise, we assume that

$$\begin{aligned} A \hbox { is VL stable}. \end{aligned}$$

#### Corollary 3.10

If $\textbf{u}^*\in C_+$ (that is, all its coordinates are strictly positive) is an equilibrium of (LotV), then $\textbf{u}^*$ is the GASS of (LotV). In other words, if the community $I=\{1,2,\ldots ,N\}$ is admissible, then $\textbf{u}^I$ is the GASS of (LotV).

#### Remark

In the previous corollary we state that *when* $\textbf{u}^*\in C_+$ *then it is the GASS of the system*. However, this is not what happens in general, specially for high–dimensional systems, in which the GASS might possess some (or even most) zero coordinates. In what follows, we *do not assume* that the GASS of the system has all positive coordinates.

Note that, since (LotV) generates a group $\mathcal {T}$, each equilibrium of $\mathcal {T}$ satisfies (2.1). If $\textbf{u}^*_\textrm{G}$ is the GASS of (LotV), we know that $\textbf{u}^*_\textrm{G}$ is a local attractor with a given basin of attraction $\mathcal {B}$ in $\mathcal {A}$. Its associated repeller is defined by

$$\begin{aligned} \mathcal {R} = \big \{\textbf{u}_0\in \mathcal {A}:\textbf{u}^*_\textrm{G} \ne \omega (\textbf{u}_0)\big \}, \end{aligned}$$

(note that for every $\textbf{u}_0\in \overline{C}_+$ the set $\omega (\textbf{x})$ is a singleton). We have the following properties:

$$\circ $$
$C_+\subset \mathcal {B}$. This follows from Proposition 3.9, since $C_+\subset C_+^{J(\textbf{u}^*_\textrm{G})}$.
$$\circ $$
$\mathcal {R}\cap \mathcal {B}=\varnothing$. In fact, if $\textbf{u}_0\in \mathcal {B}$ then it follows from Proposition 3.9 that $T(t)\textbf{u}_0\rightarrow \textbf{u}^*_\textrm{G}$.
$$\circ $$
$\mathcal {R}\cup \mathcal {B}=\mathcal {A}$. Indeed, if $\textbf{u}_0 \in \mathcal {A}$ then $\omega (\textbf{u}_0)$ is either $\textbf{u}^*_\textrm{G}$ or it is a different equilibrium of (LotV), which means that $\textbf{u}_0$ is either in $\mathcal {B}$ or in $\mathcal {R}$.
$$\circ $$
$\mathcal {R}$ is closed in $\mathcal {A}$ and, hence, $\mathcal {B}$ is open in $\mathcal {A}$. This follows from Bortolan et al. (2020, Lemma 3.27).

Also it is clear that every equilibrium of (LotV), other than $\textbf{u}^*_\textrm{G}$, is in $\mathcal {R}$.

#### Definition 3.11

Given a bounded global solution $\xi$ of (LotV), we define its **dimension** $\dim \xi$, as the number $d\in \{0,1,\ldots ,N\}$ of positive coordinates of $\xi (t)$, for any given $t\in \mathbb {R}$ (which, by invariance, does not depend on the choice of $t\in \mathbb {R}$). When $\xi$ is stationary, that is, $\xi (t)=\textbf{u}^*$ for all $t\in \mathbb {R}$ where $\textbf{u}^*$ is an equilibrium of (LotV), the dimension *d* of $\xi$ will be also referred to as the **dimension of** $\textbf{u}^*$.

#### Lemma 3.12

For any nontrivial bounded global solution $\xi$ of (LotV) in $\mathcal {B}$ we have $\alpha (\xi )\subset \mathcal {R}$.

#### Proof

Assume that $\textbf{x}\in \alpha (\xi )$ and $\textbf{x}\notin \mathcal {R}$. Hence $\textbf{x}\in \mathcal {B}$. There exists an strictly decreasing sequence $s_n\rightarrow -\infty$, with $s_n-s_{n+1}>n$, such that $\xi (s_n)\rightarrow \textbf{x}$. Since $\textbf{x}\in \mathcal {B}$ and $\mathcal {B}$ is open in $\mathcal {A}$ there exists $\eta >0$ such that $\xi (s_n)\in \overline{B}_\eta (\textbf{x})\cap \mathcal {A}\subset \mathcal {B}$. Therefore

$$\begin{aligned} d_H(T(t)(\overline{B}_\eta (\textbf{x})\cap \mathcal {A}),\textbf{u}^*_\textrm{G})\rightarrow 0 \quad \hbox { as } t\rightarrow \infty . \end{aligned}$$

Thus

$$\begin{aligned} d_H(\xi (s_n),\textbf{u}^*_\textrm{G}) = d_H(T(s_n-s_{n+1})\xi (s_{n+1}),\textbf{u}^*_\textrm{G})\rightarrow 0 \quad \hbox { as } n\rightarrow \infty , \end{aligned}$$

and since $\xi (s_n)\rightarrow \textbf{x}$, we obtain $\textbf{x}=\textbf{u}^*_\textrm{G}$. Therefore $\xi (s_n)\rightarrow \textbf{u}^*_\textrm{G}$ as $n\rightarrow \infty$. Since $\textbf{u}^*_\textrm{G}$ is a local attractor, there exists a neighborhood *U* of $\textbf{u}^*_\textrm{G}$ such that $T(t)U\subset U$ for all $t\geqslant 0$. We can choose *U* such that $\textbf{u}^*_\textrm{G}$ is a maximal invariant set in *U*. Now if $t\in \mathbb {R}$ we can choose *n* large so that $s_n<t$ and $\xi (s_n)\in U$ (since $\xi (s_n)\rightarrow \textbf{u}^*_\textrm{G}$). Thus $\xi (s)\in U$ for all $s\geqslant s_n$ and, in particular, $\xi (t)\in U$. Since this works for each $t\in \mathbb {R}$ we obtain $\xi (t)\in U$ for all $t\in \mathbb {R}$. But $\xi (\mathbb {R})\subset U$ and $\xi (\mathbb {R})$ is invariant. Since $\textbf{u}^*_\textrm{G}$ is the maximal invariant set in *U* we obtain $\xi (t)=\textbf{u}^*_\textrm{G}$ for all $t\in \mathbb {R}$, which contradicts the fact that $\xi$ is a nontrivial solution. $\square$

#### Theorem 3.13

Let $\xi$ be a bounded global solution of (LotV) in $\mathcal {B}$. Assume that there exist $\textbf{x},\textbf{u}^*\in \alpha (\xi )$ where $\textbf{u}^*$ is an equilibrium of (LotV) and $\textbf{x}\ne \textbf{u}^*$. Then

- there exist nontrivial bounded global solutions $\phi ,\psi$ in $\mathcal {A}$ such that
  $$\begin{aligned} \phi (t) \rightarrow \textbf{u}^*\hbox { as } t\rightarrow -\infty \quad \hbox { and } \quad \psi (t) \rightarrow \textbf{u}^*\hbox { as } t\rightarrow \infty , \end{aligned}$$
  with $\phi (t),\psi (t)\in \mathcal {R}$ for each $t\in \mathbb {R}$;
- $$0<\dim \textbf{u}^*<\dim \xi $$;

#### Proof

Clearly $\xi$ has to be a nontrivial solution, otherwise $\alpha (\xi )$ would be a singleton. Since $\textbf{u}^*\in \alpha (\xi )$ we have $\dim \textbf{u}^*\leqslant \dim \xi$, from the invariance of $C_+^{J(\xi (0))}$. If $\dim \textbf{u}^*= \dim \xi$, then $\textbf{u}^*$ is in $C_+^{J(\xi (0))}$ and it is the GASS of (LotV) restricted to $\overline{C_+^{J(\xi (0))}}$. This implies that there exist small neighborhoods *U* and *V* of $\textbf{u}^*$ in $C_+^{J(\xi (0))}$, with $U\subset V$ and $\textbf{x}\notin V$, such that $T(t)U\subset V$ for all $t\geqslant 0$. Since $\textbf{x},\textbf{u}^*\in \alpha (\xi )$, we can choose $s_2<s_1$ such that $\xi (s_2)\in U$ and $\xi (s_1)\notin V$. Then $\xi (s_1) = T(s_1-s_2)\xi (s_2) \in T(s_1-s_2)U\subset V$, which gives us a contradiction. The item (a) follows directly from Theorem 2.6 and Lemma 3.12.

(b) Since the stable manifold of the equilibrium $\textbf{0}=(0,\ldots ,0)$ is trivial, we cannot have $\textbf{u}^*=\textbf{0}$ hence $\dim \textbf{u}^*>0$. $\square$

#### Theorem 3.14

Let $\xi$ be a bounded global solution of (LotV) in $\mathcal {B}$ and assume that $\alpha (\xi )$ is not a singleton. Then the dimension of any equilibrium belonging to $\alpha (\xi )$ is between 1 and $d-2$, where $d=\dim \xi$.

#### Proof

From Lemma 2.2 there exists at least an equilibrium of (LotV) in $\alpha (\xi )$. Let $\textbf{u}^*$ be any such equilibrium. From the hypotheses, we can choose $\textbf{x}\ne \textbf{u}^*$ with $\textbf{x}\in \alpha (\xi )$. From Theorem 3.13 there exists a nontrivial bounded global solution $\phi$ of (LotV) in $\mathcal {R}$ such that $\phi (t)\rightarrow \textbf{u}^*$ as $t\rightarrow -\infty$ and $\phi (t)\in \alpha (\xi )$ for each $t\in \mathbb {R}$. Moreover, $0<\dim \textbf{u}^*<d$ and $\textbf{u}^*\in \mathcal {R}$. We know that every equilibria in $\mathcal {R}$ has dimension at most $d-1$. Since every equilibrium in $\mathcal {R}$ with dimension $d-1$ must be the GASS of a $(d-1)$-dimensional Lotka–Volterra system (contained in $\mathcal {R}$), the existence of $\phi$ shows that the dimension of $\textbf{u}^*$ is at most $d-2$. $\square$

By a **heteroclinic cycle** in the global attractor of (LotV) we mean collections of equilibria $\textbf{u}^*_1,\ldots ,\textbf{u}^*_m$ and nontrivial bounded global solutions $\xi _1,\ldots ,\xi _m$ of (LotV) such that, for $\textbf{u}^*_{m+1}:=\textbf{u}^*_1$, we have

$$\begin{aligned} \textbf{u}^*_i {\mathop {\longleftarrow }\limits ^{t\rightarrow -\infty }} \xi _i(t) {\mathop {\longrightarrow }\limits ^{t\rightarrow \infty }} \textbf{u}^*_{i+1}, \quad \hbox { for } i=1,\ldots ,m. \end{aligned}$$

From Bortolan et al. (2020), we know that a semigroup $\mathcal {T}$, in a metric space *X*, with a global attractor $\mathcal {A}$ and a finite collection of equilibria $\mathcal {E}=\{u^*_1,\ldots ,u^*_n\}$ is called **dynamically gradient** with respect to $\mathcal {E}$ if there are no heteroclinic cycles in $\mathcal {A}$ (in the sense of the definition above) and for each bounded global solution $\xi$ of $\mathcal {T}$ there exists $u^*_i,u^*_j\in \mathcal {E}$ such that

$$\begin{aligned} u^*_i {\mathop {\longleftarrow }\limits ^{t\rightarrow -\infty }} \xi _i(t) {\mathop {\longrightarrow }\limits ^{t\rightarrow \infty }} u^*_j. \end{aligned}$$

We say that $\mathcal {T}$ is **gradient** with respect to $\mathcal {E}$ is there exists a continuous function $V:X\rightarrow \mathbb {R}$ such that

$$\circ $$
the map $[0,\infty )\ni t \mapsto V(T(t)x)\in \mathbb {R}$ is nonincreasing for each $x\in X$;
$$\circ $$
$V(T(t)x)=V(x)$ for all $t\geqslant 0$ if and only if $x\in \mathcal {E}$.

We know from Bortolan et al. (2020, Theorem 3.41) that a semigroup $\mathcal {T}$ is gradient with respect to $\mathcal {E}$ if and only if $\mathcal {T}$ is dynamically gradient with respect to $\mathcal {E}$.

#### Corollary 3.15

Consider $N\geqslant 3$ and assume that there are no heteroclinic cycles in the global attractor of (LotV). Then, for each bounded global solution $\xi$ of (LotV), $\alpha (\xi )$ consists of a unique equilibrium of (LotV). In particular, the group generated by (LotV) is gradient with respect to its equilibria.

#### Proof

From Lemma 2.2, it follows that $\alpha (\xi )$ contains an equilibrium of (LotV), which we name $\textbf{u}^*_1$. If either $\xi$ is a stationary solution or $\dim \xi \leqslant 2$, we already know that $\alpha (\xi )=\{\textbf{u}^*_1\}$.

Assume, by contradiction, that there exists $\textbf{x}\ne \textbf{u}^*_1$ with $\textbf{x}\in \alpha (\xi )$ and that $\dim \xi \geqslant 3$. We restrict ourselves to the $(\dim \xi )$-dimensional (LotV) system of which $\xi$ is a bounded global solution, which we name (LotV) again. Thus, for this system, $\xi$ lies in the positive cone $C_+$. From Theorem 3.13 there exists a nontrivial bounded global solution $\phi$ of (LotV) in $\mathcal {R}$ such that $\phi (t)\rightarrow \textbf{u}^*_1$ as $t\rightarrow -\infty$ and $\phi (t)\in \alpha (\xi )$ for each $t\in \mathbb {R}$. We know that for some equilibrium $\textbf{u}_2^*$ of (LotV), $\phi (t)\rightarrow \textbf{u}_2^*$ as $t\rightarrow \infty$. Since $\alpha (\xi )$ is compact we obtain $\textbf{u}_2^*\in \alpha (\xi )$. Also, since $\phi$ is nontrivial and converges to $\textbf{u}^*_1$ as $t\rightarrow -\infty$, we have $\textbf{u}_2^*\ne \textbf{u}^*_1$ (for otherwise, $\phi$ would be a homiclinic solution, that is, the simplest heteroclinic cycle).

Using Theorem 3.13 for $\textbf{u}_2^*$ we obtain a nontrivial bounded global solution $\phi _2$ such that $\phi _2(t)\rightarrow \textbf{u}_2^*$ as $t\rightarrow -\infty$ with $\phi _2(t)\in \alpha (\xi )$ for each $t\in \mathbb {R}$. As before, for some equilibria $\textbf{u}_3^*$ of (LotV) we have $\phi _2(t)\rightarrow \textbf{u}_3^*$ as $t\rightarrow \infty$, $\textbf{u}_3^*\in \alpha (\xi )$, and $\textbf{u}_3^*\ne \textbf{u}_2^*$. If $\textbf{u}_3^*=\textbf{u}^*_1$ we have

$$\begin{aligned} \textbf{u}^*_1 {\mathop {\longleftarrow }\limits ^{t\rightarrow -\infty }} \phi (t) {\mathop {\longrightarrow }\limits ^{t\rightarrow \infty }} \textbf{u}_2^*{\mathop {\longleftarrow }\limits ^{t\rightarrow -\infty }} \phi _2(t){\mathop {\longrightarrow }\limits ^{t\rightarrow \infty }} \textbf{u}_3^*=\textbf{u}^*_1, \end{aligned}$$

which produces a heteroclinic cycle, and gives us a contradiction.

If $\textbf{u}_3^*\ne \textbf{u}^*_1$, we can analogously proceed with the argument. In a finite number of steps we produce a heteroclinic cycle (since there is only a finite number of equilibria) and reach a contradiction.

Thus, we proved that each bounded global solution of (LotV) converges (forward and backwards) to an equilibrium of (LotV). Therefore, the group generated (LotV) is gradient with respect to its equilibria. $\square$

#### Corollary 3.16

If $N=1$ or $N=2$, the group generated by (LotV) is gradient with respect to its equilibria.

#### Corollary 3.17

If all equilibria of (LotV) are hyperbolic and IG has no cycles, then the group generated (LotV) is gradient with respect to its equilibria. In particular, IG contains all the dynamical properties of the global attractor $\mathcal {A}$ of (LotV).

#### Proof

From Proposition 3.8 (recall that we are assuming that *A* is VL stable) it follows that IG=CG. Hence, if IG has no cycles we obtain that there are no heteroclinic cycles in the global attractor of (LotV) and the result follows from Corollary 3.15. $\square$

#### Remark 3.18

The above result allows us to formulate an algorithm that uses an a posteriori test to determine the structure of the global attractor: we need to build the IG, and test for cycles. If there are no cycles, it means that we recovered the whole structure of the global attractor, and thus we captured all the dynamics of the system.

Now assume that there exists a heteroclinic cycle

$$\begin{aligned} \textbf{u}_i^*{\mathop {\longleftarrow }\limits ^{t\rightarrow -\infty }} \xi _i(t) {\mathop {\longrightarrow }\limits ^{t\rightarrow \infty }} \textbf{u}_{i+1}^*\quad \hbox { for } i=1,\ldots ,m, \end{aligned}$$

with $\textbf{u}^*_{m+1}:=\textbf{u}_1^*$, and all the equilibria in this heteroclinic cycle are distinct. Let $W_i:=C_+^{J(\xi _i(0))}$, that is, the “wall” in which the global solution $\xi _i$ lies (which is the same for $\xi _i(t)$ for all $t\in \mathbb {R}$, due to the invariance). We have the following:

(i) $\textbf{u}_{i+1}^*$ is the GASS of $W_i$, since $\xi _i(0)\in W_i$ and $\xi _i(t)\rightarrow \textbf{u}_{i+1}^*$ as $t\rightarrow \infty$.
(ii) $$W_i\cap \overline{W_j}=\varnothing $$ for $$i\ne j$$.
(iii) $\textbf{u}_{i+1}^*\in \overline{W_i}\setminus W_i$, hence $\dim \textbf{u}_{i+1}^*< \dim W_i$. (It is clear that $\textbf{u}_{i+1}^*\in \overline{W_i}$ since $W_i\ni \xi _i(t)\rightarrow \textbf{u}_{i+1}^*$ as $t\rightarrow \infty$. On the other hand, since $W_{i+1}\ni \xi _{i+1}(t)\rightarrow \textbf{u}_{i+1}^*$ as $t\rightarrow -\infty$, we have $\textbf{u}_{i+1}^*\in \overline{W_{i+1}}$ and thus $\textbf{u}_{i+1}^*\notin W_i$.)
(iv) There can be no equilibrium in $W_i$, because if there was, $\textbf{u}_{i+1}^*$ could not be its GASS.

With this, and Corollary 3.15, we obtain the following result:

#### Theorem 3.19

If (LotV) possesses maximal frondosity, that is, all the equilibria of the system are present (or, in other words, all the communities are admissible), then there are no heteroclinic cycles, the $\alpha$-limit of every bounded global solution is a singleton and the group generated by (LotV) is gradient with respect to its equilibria.

### Ensuring maximal frondosity

We study the situation when we can ensure the maximal frondosity of the invasion graph.

#### Theorem 3.20

Assume that for every community $J\subset \{1,\ldots ,N\}$ the unique solution $\textbf{u}^*_J$ of the system

$$\begin{aligned} -A(J)\textbf{u}=\textbf{b}(J) \end{aligned}$$

has all coordinates strictly positive.

Then (LotV) has maximal frondosity, there are no heteroclinic cycles, the $\alpha$-limit of every bounded global solution is a singleton and the group generated by (LotV) is gradient with respect to its equilibria.

Furthermore, if all equilibria are hyperbolic then $I\rightarrow J$ if and only if $I\subsetneq J$.

#### Proof

As $-A(J)$ is nonsingular, the unique solution $\textbf{u}^*_J$ exists for every *J*. Since it has all coordinates positive, every community *J* is admissible and (LotV) has maximal frondosity. The conclusion comes from Theorem 3.19.

If $I\rightarrow J$, then $I\ne J$ and there exists a bounded global solution connecting $\textbf{u}^i$ and $\textbf{u}^J$. Such solution must lie in $C_+^{I\cup J}$, and hence it must converge forward to $\textbf{u}^{I \cup J}$. Hence $\textbf{u}^{I\cup J}=\textbf{u}^J$, which implies that $J = I \cup J$, that is, $I\subsetneq J$.

If $I \subsetneq J$, since all equilibria are hyperbolic, the values of $r_j(I)$ for $j\in J{\setminus } I$ must be all strictly positive. If that was not the case, that is, if $r_j(I)<0$ for some $j\in J{\setminus } I$, then $u^I$ would attract points that are in $C_+^{I \cup \{j\}}$, which is a contradiction because $u^{I \cup \{j\}}$ is the equilibrium attract all the points in $C_+^{I \cup \{j\}}$. Therefore, $I\rightarrow J$. $\square$

We discuss two situations for which the assumption of Theorem 3.20  holds. Clearly, one such case is when all intrinsic growth rates $b_j$ are positive and the interior of every positive cone $C^J_+$ is invariant under the inverse matrix $(-A(J))^{-1}$. The next result shows that such situation holds if the system is fully cooperative, that is, $a_{ij}>0$ for every $i\ne j$.

#### Lemma 3.21

Let *A* be VL stable and fully cooperative, that is $a_{ij}>0$ for every $i\ne j$. Then for every $J\subset \{1,\ldots ,n\}$ the interior of the positive cone $C^J_+$ is invariant by $(-A(J))^{-1}$, that is $(-A(J))^{-1}C^J_+\subset C^J_+$. In consequence, if only $\min _{i=1,\ldots ,N}b_i>0$ the assumption of Theorem 3.20 holds, and (LotV) has maximum frondosity.

#### Proof

We use the results of Chapter 6 of Berman and Plemmons (1994). All off diagonal entries of $-A(J)$ are nonpositive, and hence, according to the nomenclature of Berman and Plemmons (1994), page 132, we have $-A(J)\in Z^{|J|\times |J|}$. Therefore, the VL stability of *A*(*J*) is equivalent to positivity of $(-A(J))^{-1}$ (by equivalence of $H_{24}$ and $N_{38}$ in Berman and Plemmons (1994, Theorem 2.3, p. 134)). This positivity means that $(-A(J))^{-1}\overline{C^J_+}\subset \overline{C^J_+}$. But since $(-A(J))^{-1}$ maps open sets to open sets, it follows that $(-A(J))^{-1}C^J_+ = (-A(J))^{-1}\text {int}\overline{C^J_+}\subset \text {int} \overline{C^J_+} = C^J_+$, and the proof is complete. $\square$

#### Remark 3.22

Note that if all $b_i$-s are positive, the assertion of the above lemma holds under weaker assumption than full cooperativity. It is enough if we assume the inverse positivity of $-A(J)$ for every community *J*: namely that for every cone $\overline{C^J_+}$ we have $(-A(J))^{-1}\overline{C^J_+}\subset \overline{C^J_+}$.

Another case when the maximal frondosity holds, which may encompass the situation when the interaction matrix contains the negative off-diagonal entries, is a certain strong condition of diagonal dominance.

We recall that the *row norm* of a matrix $A \in \mathbb {R}^{N \times N}$ is given by

$$\begin{aligned} \Vert A\Vert _r = \max _{1 \leqslant i \leqslant N} \sum _{j=1}^N |a_{ij}|. \end{aligned}$$

#### Theorem 3.23

Assume that $\textbf{b}=(b_1,\ldots ,b_N)\in \mathbb {R}^N$ is such that $\min \limits _{i=1,\ldots ,N} b_i >0$ and that the matrix $A=(a_{ij})_{i,j=1}^N\in \mathbb {R}^{N\times N}$ is VL-stable and

$$\begin{aligned} \max _{i=1,\ldots ,N} \sum _{j \ne i} \left| \frac{a_{ij}}{a_{jj}} \right| < \frac{1}{1 + M}, \end{aligned}$$

where

$$\begin{aligned} M = \frac{\max \limits _{i=1,\ldots ,N}b_i}{\min \limits _{i=1,\ldots ,N}b_i}. \end{aligned}$$

Then the assumption of Theorem 3.20 is satisfied, and, in consequence (LotV) has maximum frondosity.

#### Proof

We choose an arbitrary community *J*. Consider the equation

$$\begin{aligned} -{A}(J) \textbf{u} = \textbf{b}(J). \end{aligned}$$

By making the change of variables $v_i=-a_{ii}u_i$ for $i\in J$, the new system has the same vector $\textbf{b}(J)\in \mathbb {R}^{|J|}$ and *A*(*J*) is replaced by the matrix $\tilde{A}=-I-P$, where $P=(p_{ij})_{i,j=1}^{|J|}$ is given by $p_{jj}=0$ and $p_{ij} = \frac{a_{ij}}{a_{jj}}$ for $i\ne j$. Note that $\Vert P\Vert _r < \frac{1}{1+M}$. We obtain the system

$$\begin{aligned} (I+P(J))\textbf{v} = \textbf{b}(J). \end{aligned}$$

Since $\Vert P(J)\Vert _r \leqslant \Vert P\Vert _r < 1$, this problem has a solution, which we call $\textbf{v}^*_J$, given by

$$\begin{aligned} \textbf{v}^*_J = (I+P(J))^{-1} \textbf{b}(J) = \sum _{n=0}^\infty (-P(J))^n \textbf{b}(J). \end{aligned}$$

Hence

$$\begin{aligned} \textbf{v}_J^*- \textbf{b}(J) = \sum _{n=1}^\infty (-P(J))^n \textbf{b}(J), \end{aligned}$$

and thus

$$\begin{aligned} \Vert \textbf{v}_J^*- \textbf{b}(J)\Vert _\infty \leqslant \frac{\Vert P(J)\Vert _r}{1-\Vert P(J)\Vert _r}\Vert \textbf{b}(J)\Vert _\infty \leqslant \frac{\Vert P\Vert _r}{1-\Vert P\Vert _r}\Vert \textbf{b}\Vert _\infty < \min _{i=1,\ldots ,N}b_i. \end{aligned}$$

This means that all entries of $\textbf{v}_J^*$ are strictly positive and the conclusion follows. $\square$

## The May–Leonard system revisited

In this section we work with the 3-dimensional (LotV) system, which can be written as

3D--LotV $$\begin{aligned} \left\{ \begin{aligned}&\dot{u}_1 = b_1u_1+a_{11}u_1^2+a_{12}u_2u_1+a_{13}u_3u_1,\\&\dot{u}_2 = b_2u_2+a_{21}u_1u_2+a_{22}u_2^2+a_{23}u_3u_2,\\&\dot{u}_3 = b_3u_3+a_{31}u_1u_3+a_{32}u_2u_3+a_{33}u_3^2. \end{aligned}\right. \end{aligned}$$

Note that for $N=3$, Theorem 3.14 shows that only one-dimensional equilibria can belong to $\alpha (\xi )$ if $\xi$ is a three-dimensional nontrivial bounded global solution of (3D–LotV) when $\alpha (\xi )$ is not a singleton. Moreover, the global solutions that belong to $\alpha (\xi )$ must be two-dimensional (otherwise, either $\textbf{0}$ or a 2-dimensional equilibrium would belong to $\alpha (\xi )$, which contradicts Theorem 3.14). When $N=1$ or $N=2$, Theorem 3.14 shows that $\alpha$-limit of any nontrivial bounded global solution can only consists of one equilibrium.

### Proposition 4.1

Consider the problem (3D–LotV) and assume that *A* is VL stable (then (3D–LotV) it has a global attractor). Assume also that there exists a bounded global solution $\xi$ such that $\alpha (\xi )$ is not a singleton. Then (3D–LotV) has exactly 5 equilibria, namely $\textbf{0}$, three 1-dimensional equilibria $\textbf{u}_1^*$, $\textbf{u}_2^*$ and $\textbf{u}_3^*$, and one 3-dimensional equilibrium $\textbf{u}^*_\textrm{G}$ (the GASS of the system). Moreover, $\alpha (\xi )$ is precisely a heteroclinic cycle connecting $\textbf{u}_1^*$, $\textbf{u}_2^*$ and $\textbf{u}_3^*$.

### Proof

Firstly, note that if either $\dim \xi <3$ or $\xi$ is stationary, $\alpha (\xi )$ is a singleton. Hence $\xi$ is a nontrivial bounded global solution with $\dim \xi =3$, and thus it is in $C_+$. We refine the reasoning of Corollary 3.15. Let $\textbf{u}_1^*\in \alpha (\xi )$ and $\textbf{x}\in \alpha (\xi )$ be such that $\textbf{x}\ne \textbf{u}_1^*$. We know, from Theorem 3.14 that $\dim \textbf{u}_1^*=1$, and from Theorem 3.13 there exists a nontrivial bounded global solution $\phi _1$ of (3D–LotV) such that $\phi _1(t)\rightarrow \textbf{u}_1^*$ as $t\rightarrow -\infty$. Furthermore, we can see that $\phi _1$ must be two-dimensional.

Let $\textbf{u}_2^*$ be the GASS of $W_1:=\overline{C_+^{J(\phi _1(0))}}$ (note that $\dim W_1=2$). Since $\textbf{u}_2^*\in \alpha (\xi )$ we know that $\dim \textbf{u}_2^*=1$. We know that (3D–LotV) restricted to $W_1$, which is two-dimensional, is gradient (see Corollary 3.16) and, hence, $\textbf{u}^*_2\ne \textbf{u}^*_1$. Moreover, $W_1$ does not have a two-dimensional equilibrium, for otherwise $\textbf{u}_2^*$ would not be the GASS of $W_1$.

Applying Theorem 3.13 for $\textbf{u}_2^*$ we obtain a nontrivial 2-dimensional bounded global solution $\phi _2$ such that $\phi _2(t)\rightarrow \textbf{u}_2^*$ as $t\rightarrow -\infty$. As before, there exists an equilibrium $\textbf{u}_3^*$, the GASS of $W_2:=\overline{C_+^{J(\phi _2(0))}}$, such that $\phi _2(t)\rightarrow \textbf{u}_3^*$ as $t\rightarrow \infty$, $\textbf{u}_3^*\ne \textbf{u}_2^*$ and $\textbf{u}_3^*\in \alpha (\xi )$. As before, $\dim \textbf{u}_3^*=1$ and $W_2$ does not have a 2-dimensional equilibrium. If $\textbf{u}_3^*=\textbf{u}_1^*$ then $W_1=W_2$ and we would have a heteroclinic cycle of (3D–LotV) in $W_1$, which cannot happen since $\dim W_1=2$. Thus $\textbf{u}_3^*\ne \textbf{u}_1^*$ and $W_1\ne W_2$.

Applying Theorem 3.13 once again, now for $\textbf{u}_3^*$, we obtain a nontrivial 2-dimensional bounded global solution $\phi _3$ such that $\phi _3(t)\rightarrow \textbf{u}_3^*$ as $t\rightarrow -\infty$. Thus, if $\textbf{u}^*$ is the GASS of $W_3:=\overline{C_+^{J(\phi _3(0))}}$, we have $\dim \textbf{u}^*=1$, $\textbf{u}^*\ne \textbf{u}^*_3$ and $W_3$ does not have 2-dimensional equilibria. If $\textbf{u}^*=\textbf{u}_2^*$ then $W_2=W_3$ and there would be a heteroclinic cycle of (3D–LotV) in $W_2$, which cannot happen. Hence $\textbf{u}^*=\textbf{u}_3^*$, and none of the three walls have 2-dimensional equilibria. Moreover, $\phi _1,\phi _2,\phi _3$ constitutes a heteroclinic cycle in $\alpha (\xi )$ connecting the three 1-dimensional equilibria of (3D–LotV).

Note that, since the three 1-dimensional equilibria have nontrivial unstable manifolds, neither of them can be the GASS of the system in 3-dimensions. Therefore, (3D–LotV) must have a three-dimensional equilibrium which is the GASS.

Lastly, assume that there exists $\textbf{x}\in \alpha (\xi )$ which is not in the cycle defined by $\phi _1$, $\phi _2$, $\phi _3$. Then $\textbf{x}$ cannot belong $W_i\setminus \operatorname {int}W_i$, $i=1,2,3$, because then, as $\textbf{x}$ is not an equilibrium, we would have $\alpha (\textbf{x}) = \{0\}$, and zero cannot belong to $\alpha (\xi )$. This means that $\textbf{x}\in \operatorname {int}W_i$ for some $i=1,2,3$. Then $\omega (\textbf{x})=\{\textbf{u}^*_{i+1}\}$, since $\textbf{u}^*_{i+1}$ is the GASS of $W_i$ (here $\textbf{u}^*_4:=\textbf{u}^*_1$), and $\alpha (x)=\{\textbf{u}^*_i\}$ since by Lemma 3.14 in two dimensions $\alpha$ limit must be a singleton. This means that $\textbf{x}$ belongs to a bounded global solution connecting $\textbf{u}^*_i$ and $\textbf{u}^*_{i+1}$. But, since the unstable manifold of a one dimensional equilibrium on a two dimensional wall must be one dimensional, the solution that connects the two equilibria $W_i$ is unique, namely $\phi _i$. Hence, $\textbf{x}=\phi _i(t)$ for some $t\in \mathbb {R}$. $\square$

**Fig. 1:**
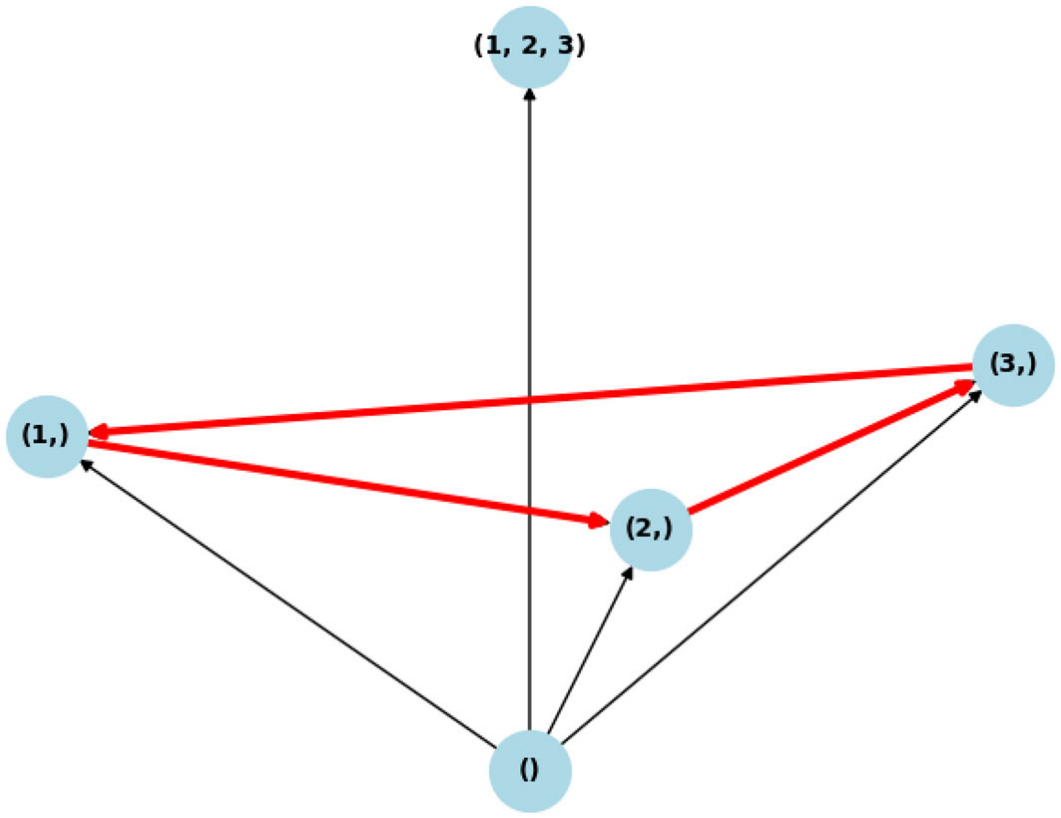
Invasion graph for the May–Leonard or for the Rock-Scissors-Paper Lotka–Volterra System, which is the only possible cyclic invasion graph Lotka–Volterra systems in dimension 3 with a VL stable matrix

The most well-known cyclic invasion graph for Lotka–Volterra systems with VL stable matrix *A* is the May–Leonard model May and Leonard (1975), see Fig. 1 for its IG. This invasion graph can be generated taking all intrinsic growth rates equal to 1, and the matrix *A* being:

$$\begin{aligned} A = \begin{pmatrix} -1 & -\beta & -\alpha \\ -\alpha & -1 & -\beta \\ -\beta & -\alpha & -1 \end{pmatrix}, \end{aligned}$$

with $0<\alpha< 1 < \beta$, $\alpha \beta < 1$, and $\alpha +\beta < 2$, see May and Leonard (1975). The same invasion graph is given by the matrix $A^{(3)}_d$ below, with $d\in (-1, 0)$, which is more clearly a matrix that represents a Rock-Scissors-Paper dynamics:

$$\begin{aligned} A^{(3)}_d = \begin{pmatrix} d & -1 & 1 \\ 1 & d & -1 \\ -1 & 1 & d \end{pmatrix}. \end{aligned}$$

### Theorem 4.2

For (3D–LotV), the only VL stable case in which there exists a heteroclinic cycle is the May–Leonard.

### Proof

The only way to have a cycle in 3*D* is if some nontrivial 3-dimensional bounded global solution $\xi$ has a nontrivial $\alpha$-limit set $\alpha (\xi )$. From the previous theorem, there are no 2-dimensional equilibria and $\alpha (\xi )$ consists of exactly a cycle connecting the three 1-dimensional equilibria, and the system has a 3-dimensional GASS. It is simple to see that $\alpha (\xi )$ is the unique heteroclinic cycle of (3D–LotV), since the bounded global solutions connecting the 1-dimensional equilibria are unique. Thus, it follows that every other nontrivial 3-dimensional bounded global solution $\psi$ of (3D–LotV) either have $\alpha (\psi )=\{\textbf{0}\}$ or $\alpha (\psi )=\alpha (\xi )$. In either case $\omega (\psi )$ consists of the GASS of (3D–LotV). $\square$

**Fig. 2:**
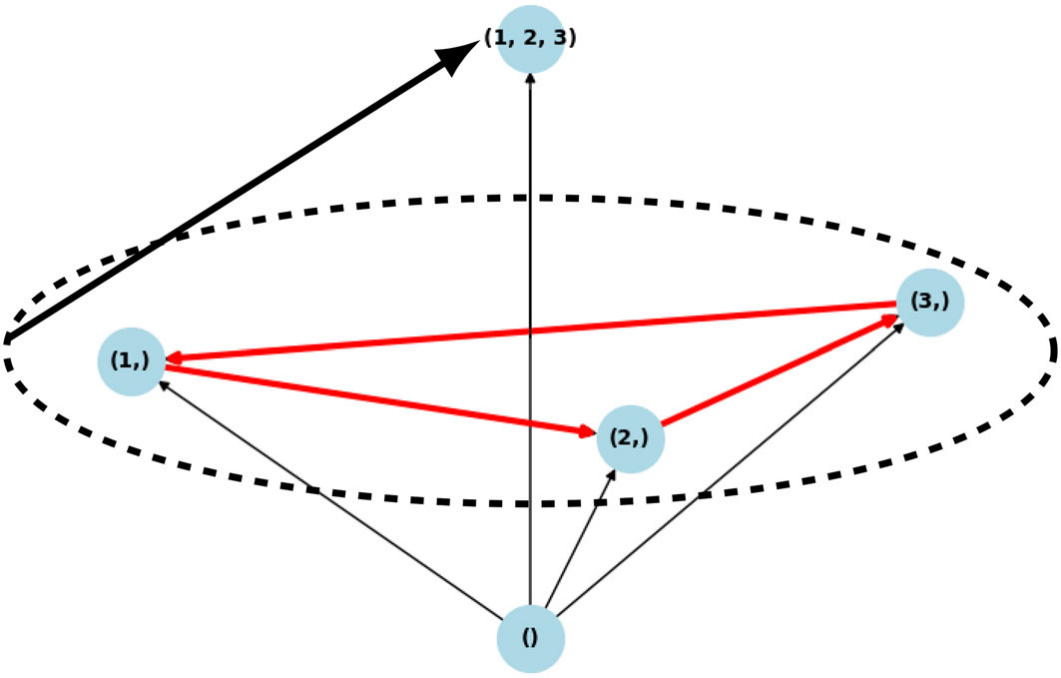
Structure of the global attractor for the May–Leonard case. Apart from the edges from Fig. 1, we obtain the additional hyperedge $\{ (1,) (2,) (3,) \} \rightarrow (1,2,3)$ that represent those solutions whose $\omega$-limit is the GASS and $\alpha$-limit is the cycle of one species equilibria

In order to express the structure of the global attractor for the VL stable case of May–Leonard dynamics, the notion of graph between the equilibria is insufficient. One possibility is to use the concept of *hypergraph*, i.e. the pair (*V*, *E*), where *V* are vertices, and $E\subset P(V)\times P(V)$ (*P*(*V*) being the family of nonempty subsets of *V*), i.e. edges do not exist only between vertices, but also between sets of vertices, we call such edges hyperedges Ferraz de Arruda et al. (2024). The structure of the global attractor of the May–Leonard case is depicted in Fig. 2.

We formulate an open question for the general case

**Open question.** From our previous analysis we know that for every hyperedge, its target must be a singleton (as the matrix *A* is VL stable), and its source, if it is not a singleton, must contain a cycle. We leave open the question: how to extend the IG algorithm to construct all such hyperedges for the *N* dimensional case with cycles?

A matrix $A=(a_{ij})_{i,j=1}^N \in \mathbb {R}^{N\times N}$ is said to be:

$$\circ $$
**strongly row diagonally dominant** if for each $i=1,\ldots ,N$
  $$\begin{aligned} |a_{ii}| > \sum _{j\ne i} |a_{ij}|; \end{aligned}$$
$$\circ $$
**strongly column diagonally dominant** if for each $j=1,\ldots ,N$
  $$\begin{aligned} |a_{jj}| > \sum _{i\ne j} |a_{ij}|. \end{aligned}$$

### Corollary 4.3

If *A* is either row diagonally dominant or column diagonally dominant, there are no heteroclinic cycles in (3D–LotV). In general, for a Lotka–Volterra system (LotV) in any dimension, if *A* is either row or column diagonally dominant, then there is no cycle involving only single-species equilibria.

### Proof

Let us assume that we have a cycle of connections between one-species equilibria in arbitrary multidimensional dimensional situation. In 3D case, as it is shown in Theorem 4.2, this is the only possible cycle. Assume that the cycle has the form that is, $\textbf{u}_1^* \rightarrow \textbf{u}_2^* \rightarrow \textbf{u}_3^* \rightarrow \ldots \textbf{u}_K^* \rightarrow { \textbf{u}_{K+1}^*:=}\textbf{u}_1^*$ (we can assume without loss of generality that this is the order of the cycle by simply renaming the species, if necessary). The invasion rates are given by:

$$\begin{aligned} r_{i}(\textbf{u}_{i+1}^*) = b_{i} - a_{i, i+1} \frac{b_{i+1}}{a_{i+1, i+1}} \hbox { for } i=1,\ldots , K, \end{aligned}$$

where $$a_{K,K+1}:=a_{K,1}$$.

*Claim:* All these invasion rates are nonpositive.

We prove that $r_1(\textbf{u}_2^*) \leqslant 0$ and the claim for other invasion rates holds analogously. Since there is a connection from $\textbf{u}_1^*$ to $\textbf{u}_2^*$, then $\textbf{u}_2^*$ must be the GASS of the two-dimensional problem involving species 1 and 2. If $r_1(\textbf{u}_2^*) > 0$, the equilibrium $\textbf{u}_2^*$ would be unstable, which is a contradiction. Thus, the claim is proved.

Therefore, we have:

$$\begin{aligned} b_i \leqslant a_{i, i+1} \frac{b_{i+1}}{a_{i+1,i+1}} \hbox { for } i=1,\ldots ,K. \end{aligned}$$

Since all equilibria $\textbf{u}_i^*$ exist, it must be $b_i>0$. Multiplying the *K* inequalities above by each other, we obtain

$$\begin{aligned} 0< \prod _{i=1}^K b_i \leqslant \frac{\prod _{i=1}^K a_{i,i+1}}{\prod _{i=1}^K a_{i,i}} \prod _{i=1}^K b_i, \end{aligned}$$

which implies that

$$\begin{aligned} 1 \leqslant \frac{\prod _{i=1}^K|a_{i,i+1}| }{\prod _{i=1}^K |a_{i, i}| }, \end{aligned}$$

and contradicts either the row or column diagonal dominance of *A*.

Note that if all equilibria in the cycle are assumed to be hyperbolic, then the inequalities in diagonal dominance conditions can be relaxed to be non-strict. $\square$

## Computational results

### Constructing and detecting Volterra–Lyapunov stability

Our method to study cycles in ecological systems was then to generate VL stable matrices and calculate the associated invasion graphs by Algorithm 3.6. The VL stable matrices can be generated randomly using Theorem 3.2, following the steps:

$$\circ $$
Generate a random positive diagonal matrix *H*.
$$\circ $$
Generate a random antisymmetric matrix *J*: to do this, we must generate a random matrix *K* and then take $J = K - K^T$.
$$\circ $$
Finally, we generate a random symmetric stable matrix *S*: to do this, we generate a random matrix *F*, and then we take the symmetric stable matrix $S = F + F^T - \alpha I$, where $\alpha \geqslant 0$ is big enough so that *S* has only strictly negative eigenvalues.

With a generator of random VL stable matrices, we can study computationally the usual dynamics of Volterra–Lyapunov stable Lotka–Volterra systems. This greatly improved our ability to understand the dynamics for VL stable matrix *A*, because contrary to “naive” method consisting in random generation of a matrix *A*, and checking if *A* is VL stable, with this new method, the constructed matrices *A* are always VL stable.

Constructing VL stable matrices helps us to understand what possible behaviors we have for the global attractors for problem (LotV) with such interaction matrices. There is however, another big problem in applications: given a matrix obtained by an abstract or numerical model, how can we verify if this matrix is Volterra–Lyapunov stable, so that we can apply the results in Takeuchi (1996) and guarantee that every submatrix of *A* generates a dynamics with a globally stable asymptotic equilibrium? Several articles provide mathematical methods to check if a matrix is VL stable (Kraaijevanger 1991; Redheffer 1985; Cross 1978), and even models not related to ecology can be studied using Volterra–Lyapunov stability (Mayengo 2023; Chien and Shateyi 2021), if VL-stability is concluded.

Here, we present a numerical approach to discover if a matrix *A* is Volterra–Lyapunov stable. The great difficulty in this problem is finding an appropriate Volterra multiplier *H*, a diagonal positive matrix such that $HA+A^TH$ is a symmetric stable matrix (all eigenvalues are strictly negative).

Randomly sampling *H*, and verifying if $HA+A^T H$ is stable, is very costly in terms of time, specially for higher dimensions, and not very conclusive without running over many possible multipliers.

Let $\mathcal {V}$ be the space of diagonal matrices in $\mathbb {R}^N$ with all elements $d_i \in (0,1)$. A matrix *A* is VL stable if, and only if, there exists a matrix $H \in \mathcal {V}$ such that $HA + A^T H$ is stable. Our approach is to define an objective function $\mathcal {O}:\mathcal {V} \rightarrow \mathbb {R}$ as follows:

$$\begin{aligned} \mathcal {O}(H) \hbox { is the maximum of the eigenvalues of the matrix } H A + A^T H. \end{aligned}$$

The problem of finding out if *A* is VL stable reduces to the minimization of function $\mathcal {O}$ over $\mathcal {V}$ and checking whether this function attains some strictly negative value.

We use the function *minimize* from Python’s library scipy.optimize, to minimize the objective function $\mathcal {O}$. The function stops if it finds $H \in \mathcal {V}$ such that $\mathcal {O}(H)$ is strictly negative (below a negative threshold like $-10^{-10}$), and in this case the program concludes that the matrix *A* is VL stable. Clearly, the minimization process can stop in local minima, so that it is appropriate to try the minimization inside a loop so that hundreds or thousands of initial points $H\in \mathcal {V}$ are used as starting points of the optimization process. If in the end of all the minimization trials, no $H \in \mathcal {V}$ is found so that $\mathcal {O}(H)$ is below a negative threshold, the result of the test is inconclusive.

This test has a $100\%$ precision because it only concludes that a matrix is VL stable if it really is. The problem is that the result can be inconclusive because the minimization can get stuck in local minima and not achieve the global minimum of function $\mathcal {O}$, which could be negative or not. To mitigate this problem, we can try thousands of different initial conditions $H \in \mathcal {V}$. We denote by $n_{initial}$ the number of initial values of *H* with which we run the minimization process before ending the test. For any VL stable matrix *A*, there is some $H \in \mathcal {V}$ such that $HA + A^T H$ is stable, and by continuity this implies that there exists an open and not empty set $\mathcal {J} \subset \mathcal {V}$ such that for any $H \in \mathcal {J}$, $\mathcal {O}(H) < 0$. Since the volume of $\mathcal {J}$ in $\mathcal {V}$ is strictly positive, the probability of not concluding VL-stability for a VL stable matrix *A* can be made arbitrarily small if we try sufficiently many well distributed initial values for *H* (as $n_{initial} \rightarrow \infty$).

**Fig. 3:**
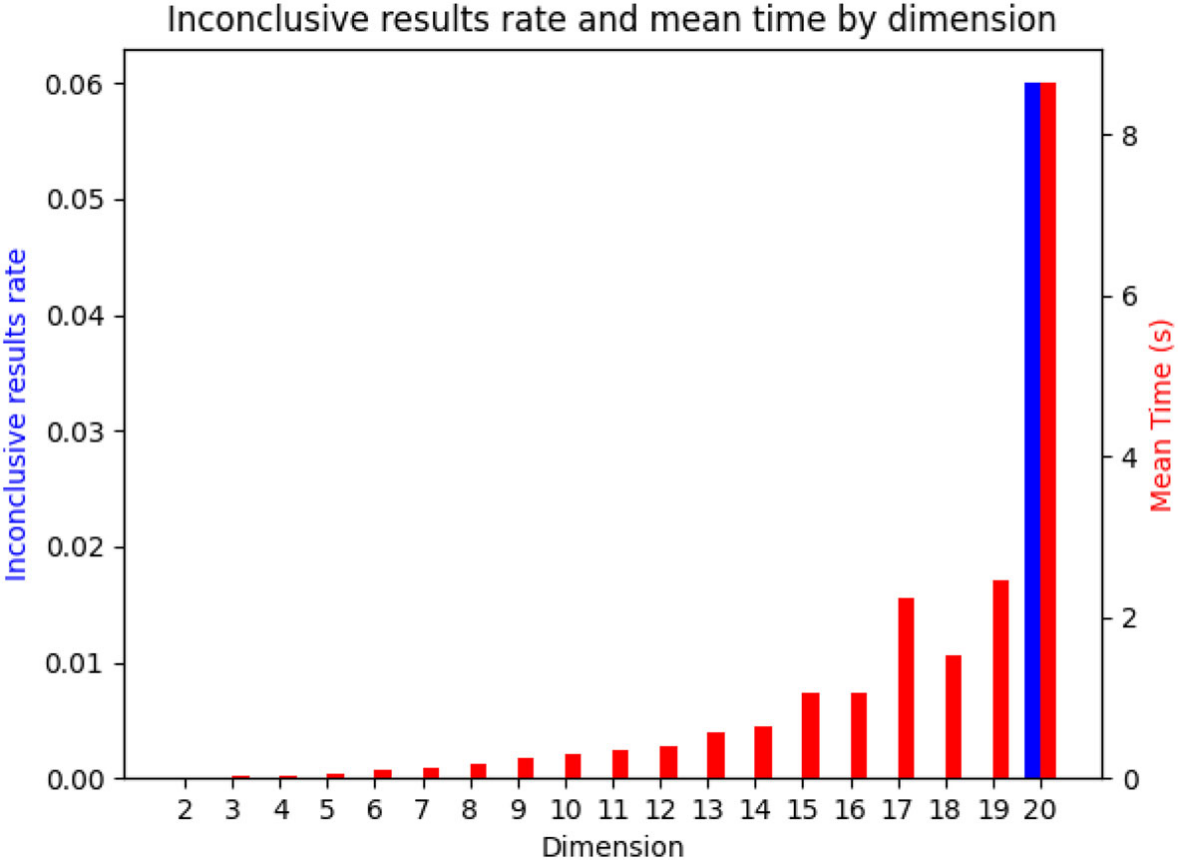
Effectiveness and speed of the algorithm for testing Volterra–Lyapunov stability. A total of 50 random VL stable matrices were passed through the program that tried minimizing function $\mathcal {O}$ with 50 different initial values of *H*. The inconclusive rate denotes the rate of VL stable matrices that the algorithm could not detect. The algorithm detected VL stability for all the matrices with dimension up to 19, which means a $100\%$ recall, and it failed only for $6\%$ of the matrices in dimension 20. The mean time to run the algorithm was always less than 10 seconds

Moreover, the test is very fast and works for any dimension. For dimension between 2 and 20, we can see below that the algorithm takes only some seconds to run and detects almost $100\%$ of Volterra–Lyapunov stable matrices when we use $n_{initial} = 50$. See the results in Fig. 3. If we have at our disposal the exact computational algorithm for finding the largest eigenvalue of a symmetric matrix, we can formulate the algorithm that determines whether a matrix is VL stable. If a given matrix is VL stable, then the algorithm halts, but if it is not VL stable, then the algorithm does not halt. Therefore, we prove that the question of the matrix VL stability is partially decidable. Note that the set $\mathcal {V}$ is open in the separable space $\mathbb {R}^N$ and therefore is is possible to find a countable dense set $\{H_i\}_{i=1}^\infty \subset \mathcal {V}$. We formulate the following algorithm:

Initialize $$i:=1$$.

- Find the largest eigenvalue $\lambda _i$ of $H_iA+A^TH_i$.
- If $\lambda _i < 0$ then *A* is VL stable. Stop.
- Otherwise increment *i* and return to step **(a)**.

#### Theorem 5.1

If *A* is VL stable then the above algorithm halts.

#### Proof

If there exists $H\in \mathcal {V}$ such that all eigenvalues of $HA+A^TH$ are negative then, as the eigenvalues depend continuously on the matrix entries, there exists $H_i$ with the same property and the algorithm halts. $\square$

### Cyclic behavior

This section presents two examples of cyclic behavior in the system (LotV) with a VL-stable matrix, which can be viewed as a generalization of May–Leonard-type dynamics to higher dimensions. To the best of our knowledge, these examples have not been studied previously. So, generalizing the May–Leonard example from May and Leonard (1975), illustrated in the Fig. 1, which represents a cycle of the form Rock–Paper–Scissors, we construct an example of a Volterra–Lyapunov stable matrix $A^{(5)}_d$ that induces a dynamics of the form Rock–Paper–Scissor–Lizard–Spock (which we name RPSLS, for short) Kass and Bryla (2009). The intrinsic growth rates were all chosen to be 1, and we created the matrix $A^{(5)}_d$ based on Proposition 3.2. The matrix *H* is taken as identity, and $A^{(5)}_d = S_d + J$, where $S_d$ is a diagonal matrix with diagonal $d < 0$, and *J* is an antisymmetric matrix representing the cyclic dynamics, more precisely, we get:

$$\begin{aligned} A^{(5)}_d = \begin{pmatrix} d & -1 & 1 & -1 & 1 \\ 1 & d & -1 & 1 & -1 \\ -1 & 1 & d & -1 & 1 \\ 1 & -1 & 1 & d & -1 \\ -1 & 1 & -1 & 1 & d \end{pmatrix} \end{aligned}$$

**Fig. 4:**
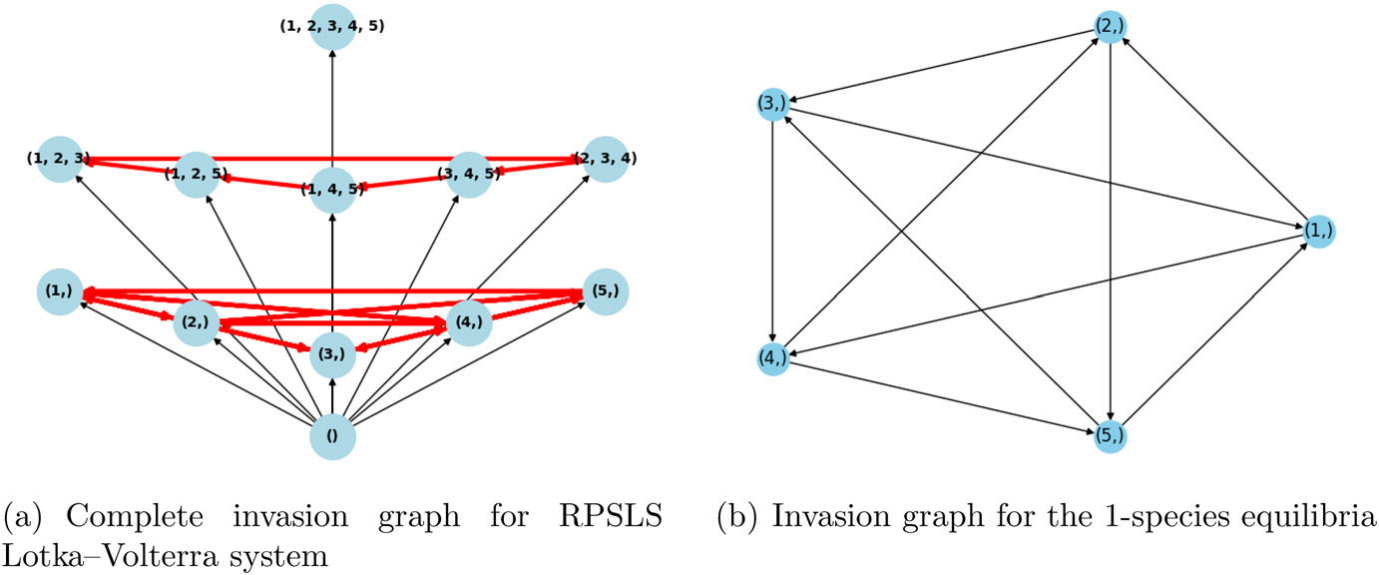
The RPSLS dynamics in a Volterra–Lyapunov system of equations given by matrix $A^{(5)}_d$ mentioned above with $d\in (-1,0)$. The nodes represent admissible equilibria, (node (1,2,3), for example, is the equilibrium with species 1, 2 and 3 coexisting). The arrows represent transitions between equilibria, which happen in the global attractor of the dynamical system (LotV). The cycles are represented in red. Notice the cycle of size five connecting all the one species equilibria and the other cycle of size five connecting all the three species equilibria. Since all equilibria are hyperbolic the graph in Fig. 4a represents all connections in the global attractor

**Fig. 5:**
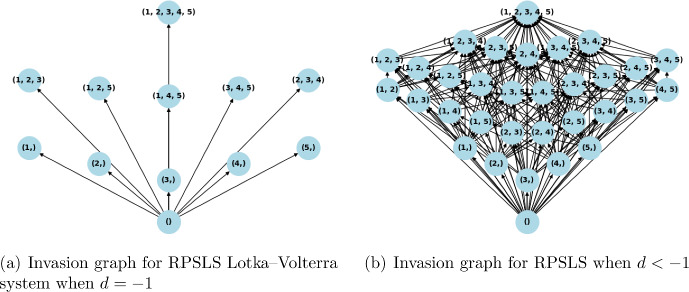
The RPSLS dynamics with high self-regulation ($d\leqslant -1$). When $d = -1$, the graph is the same as before, but without the cycles’ transitions. When $d < -1$, we have the bifurcation of several equilibria (all communities become admissible), and the structure of the graph is the same as the structure of the symmetric decoupled system with the diagonal matrix $A_S(d)$ (the symmetric part of $A^{(5)}_d$). Since the system for $d=-1$ contains nonhyperbolic equilibria, there may exist heteroclinic connections not visible in graph in Fig. 5a. The graph depicted in Fig. 5b is acyclic and all equilibria are hyperbolic, whence all connections are present in the graph, and in fact the whole structure of the global attractor is recovered

In Fig. 1, we can see the Invasion graph for the May–Leonard system, that coincides with the invasion graph of the Rock–Paper–Scissors system generated by matrix $A^{(3)}_d$. In Fig. 4a we can see the invasion graph of the RPSLS Lotka–Volterra system generated by $A^{(5)}_d$:

The increase of the absolute value of the diagonal in matrix $A^{(5)}_d$, makes the system more diagonally dominant, and we observe the inhibition of the cycle behavior. In this case, this inhibition is very illustrative. The cycles exist in the invasion graph when $d\in (-1, 0)$, they cease to exist when $d = -1$ (the case of non-hyperbolicity), and when $d < -1$, we have observed by simulation that the invasion graph determined by matrix $A^{(5)}_d$ is the same as the invasion graph determined by the diagonal matrix $A_S(d)$, which does not have any cycles because of the symmetry of $A_S(d)$ (symmetric Lotka–Volterra systems have a Lyapunov function Pykh (2001)). This graph determined by $A^{(5)}_d$ when $d<-1$ contains all possible communities as admissible communities, and has a gradient structure (Fig. 5).

**Fig. 6:**
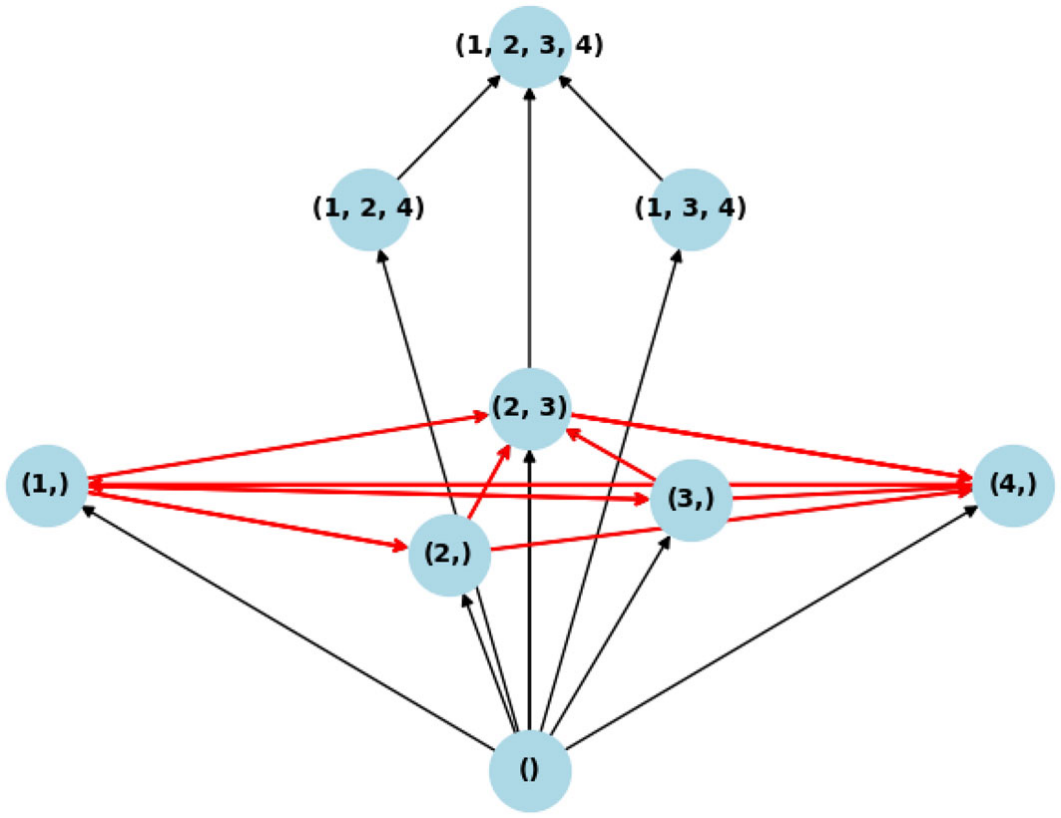
Invasion graph for the matrix *A* with a cycle containing a double species invasion in a single species environment. All equilibria of the system are hyperbolic, and therefore all connections in the global attractor are captured in the graph

It was proven in Theorem 4.2 that the only cyclic invasion graph for VL stable Lotka–Volterra systems in 3D is the May–Leonard example. In the previous example, we showed that for VL stable matrix *A*, there can be cycles involving equilibria with coexisting species, with size 5, and this example can be generalized to prove that there can exist cycles of arbitrary size. Now we show that the cycles can connect equilibria with different number of species coexisting in each. This can be done using a VL stable matrix constructed with Proposition 3.2, with *S* being a negative diagonal matrix, and *J* reflecting the fact that species 1 is replaced by species 2 and 3, which are both replaced by species 4, which is replaced again by species 1. More specifically, we use (with $d = -0.5$) (Fig. 6):

$$\begin{aligned} A = \begin{pmatrix} d & \quad -1 & \quad -1 & \quad 1 \\ 1 & \quad d & \quad 0 & \quad -1 \\ 1 & \quad 0 & \quad d & \quad -1 \\ -1 & \quad 1 & \quad 1 & \quad d \end{pmatrix} \end{aligned}$$

We now try to generalize the comment that increasing the diagonal of the matrix *A* inhibit cyclic behavior. We first observe that the interactions matrix *A* can be decomposed in a symmetric and an antisymmetric part, respectively:

$$\begin{aligned} A_S = \frac{A + A^T}{2} \quad \hbox { and } \quad A_J = \frac{A - A^T}{2}. \end{aligned}$$

The whole interactions matrix is given by:

$$\begin{aligned} A = A_S + A_J. \end{aligned}$$

**Fig. 7:**
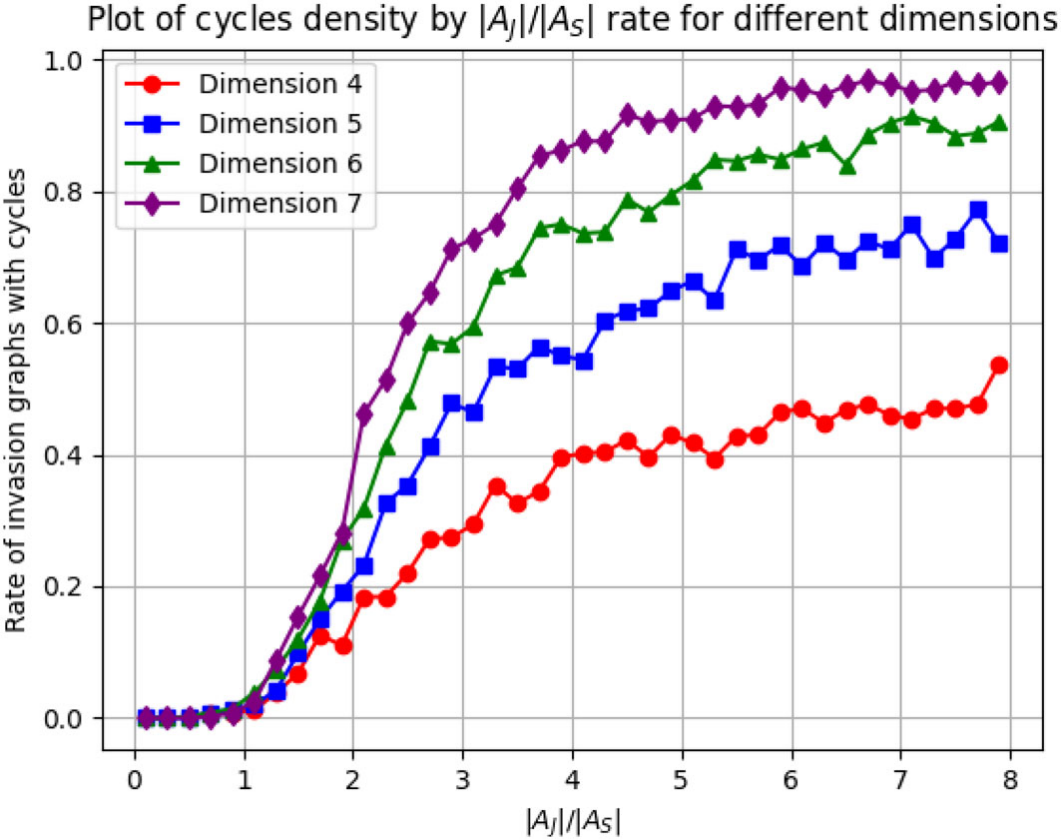
Cycle density vs. antisymmetric by symmetric Rate. The norm of the matrix was taken as the sum of its absolute values. We randomly sampled 500 matrices *A* for each given rate $|A_J|/|A_S|$ and dimension, and calculated the associated invasion graphs. The y-axis represents the rate of those 500 invasion graphs that contained cycles

If $| \cdot |$ denotes the sum of the absolute values of the entries of a matrix, we observed that the presence of cycles in the invasion graph of Lotka–Volterra equations with interactions matrix *A* is directly proportional to $|A_J|$, and inversely proportional to $|A_S|$. In other words, antisymmetric interactions facilitate the existence of cycles, while symmetric interactions inhibit them (Fig. 7).

We call into attention three aspects of this result.

$$\circ $$
The cycle’s density goes to zero when the antisymmetric part tends to zero, and the interaction’s matrix becomes symmetric. This is in accordance with the fact that symmetric Lotka–Volterra systems are gradient (see Pykh (2001)).
$$\circ $$
Additionally, we sampled millions of row diagonally dominant or column diagonally dominant matrices, allowing positive and negative intrinsic growth rates and interactions, and no cyclic invasion graph was found. This made us very confident that diagonally dominant interaction matrices eliminate the possibility of heteroclinic cycles.
$$\circ $$
The self-regulation rates $a_{ii}$, $i\in \{1, \dots , n\}$ are in the symmetric part of matrix *A*, that is, the diagonal of $A_S$ is the diagonal of *A*, and the diagonal of $A_J$ is zero. Therefore, when the matrix *A* is diagonally dominant, the symmetric part of *A* is greater than its antisymmetric part, so the diagonally dominant matrices are located in the region where $|A_J| / |A_S| < 1$, which is in accordance with the conjecture that diagonally dominant matrices have no cycles. We now understand that the absence of cycles is not only caused by diagonal dominance, but, more generally, it is caused by the strength of symmetric relations.
$$\circ $$
Finally, an ecological system has a high probability of containing cycles when the symmetric relations are weak in relation to the antisymmetric relations, specially for systems with a lot of species.

We finish with an open questions regarding our computational experiments:

**Open question 1.** Can we prove mathematically that there are no cycles in the global attractor of Lotka–Volterra systems with (row or column) diagonally dominant interaction matrix *A*?

One could also propose a stronger conjecture, which may serve as an interesting starting point for further research.

**Open question 2.** Can we prove mathematically that there are no cycles in the global attractor of Lotka–Volterra systems with the matrix *A* with (appropriately defined) dominating symmetric part?
